# The Autism Palette: Combinations of Impairments Explain the Heterogeneity in ASD

**DOI:** 10.3389/fpsyt.2020.503462

**Published:** 2020-12-02

**Authors:** Ábel Fóthi, Latha Soorya, András Lőrincz

**Affiliations:** ^1^Institute of Enzymology, Research Centre for Natural Sciences, Budapest, Hungary; ^2^Department of Artificial Intelligence, Faculty of Informatics, Eötvös Loránd University, Budapest, Hungary; ^3^Department of Psychiatry and Behavioral Sciences, Rush Medical College, Chicago, IL, United States

**Keywords:** neuropsychiatric disorder, cognition, social behavior, genetics, dimensionality, reward, autoencoder, component

## Abstract

Autism spectrum disorder (ASD) is a heterogeneous neuropsychiatric condition traditionally defined by core symptoms in social behavior, speech/communication, repetitive behavior, and restricted interests. Beyond the core symptoms, autism has strong association with other disorders such as intellectual disability (ID), epilepsy, schizophrenia among many others. This paper outlines a theory of ASD with capacity to connect heterogeneous “core” symptoms, medical and psychiatric comorbidities as well as other etiological theories of autism in a unifying cognitive framework rooted in neuroscience and genetics. Cognition is embedded into an ever-developing structure modified by experiences, including the outcomes of environment influencing behaviors. The key constraint of cognition is that the brain can handle only 7±2 relevant variables at a time, whereas sensory variables, i.e., the number of sensory neurons is orders of magnitude larger. As a result, (a) the extraction, (b) the encoding, and (c) the capability for the efficient cognitive manipulation of the relevant variables, and (d) the compensatory mechanisms that counteract computational delays of the distributed components are critical. We outline our theoretical model to describe a Cartesian Factor (CF) forming, autoencoder-like cognitive mechanism which breaks combinatorial explosion and is accelerated by internal reinforcing machineries and discuss the neural processes that support CF formation. Impairments in any of these aspects may disrupt learning, cognitive manipulation, decisions on interactions, and execution of decisions. We suggest that social interactions are the most susceptible to combinations of diverse small impairments and can be spoiled in many ways that pile up. Comorbidity is experienced, if any of the many potential impairments is relatively strong. We consider component spoiling impairments as the basic colors of autism, whereas the combinations of individual impairments make the palette of autism. We put forth arguments on the possibility of dissociating the different main elements of the impairments that can appear together. For example, impairments of generalization (domain general learning) and impairments of dealing with many variable problems, such as social situations may appear independently and may mutually enhance their impacts. We also consider mechanisms that may lead to protection.

## Introduction

Individuals with ASD have considerable symptom heterogeneity, but only a few core symptoms: impairments in social interaction and communication as well as restricted and repetitive behaviors. While many of the ASD symptoms appear in the non-social domain, those are not core symptoms. In addition, variability of IQ is remarkable. Although ID (IQ < 70) is a common comorbid condition in ASD and the mean IQ of autistic individuals is below 100, many individuals have normal or above average IQ (1). Moreover, only a proportion of people with ID are diagnosed with ASD. In turn, core ASD symptoms are not the strict consequence of low IQ. This high variability in general cognitive ability is central to our unifying neurocognitive theory of ASD. In addition, we ask what makes emotion recognition and social interaction hard for high IQ individuals?

Specifically, we propose that ASD results from a combination of (i) impairments that corrupt the solution of cognitive problems having hidden variables^1^ (the higher the number and complexity of the hidden variables the larger the corruption) and (ii) emotional conditioning together. In our theory, social cognitive processes are viewed as a particularly vulnerable cognitive mechanism due to the high complexity of social interactions, since such interactions inherently have a large number of hidden variables. Furthermore, in order to estimate the intentions that will guide future actions, social cognitive processes need to deal with internal emotions, the emotions of the partner(s) and information that is emerging from shared experiences of the individuals.

Sensory processing is the key in our considerations. A plethora of sensory neurons give rise to huge space where sensed episodes happen, since the dimension of the space equals the number of sensors and the size of the space is scales with that number in the exponent. From the computational aspect the space is overwhelming, and the general task is at odds with any computational power, not to mention our cognitive capacity that can only deal with 7 ± 2 items at a time (2). If the dimensionality of the space cannot be reduced adequately, then searching for solutions to problems and executive functions become troublesome, or sometimes impossible. We propose that reduction of the dimensionality happens to some extent in ASD in various ways.

**Figure 1:**
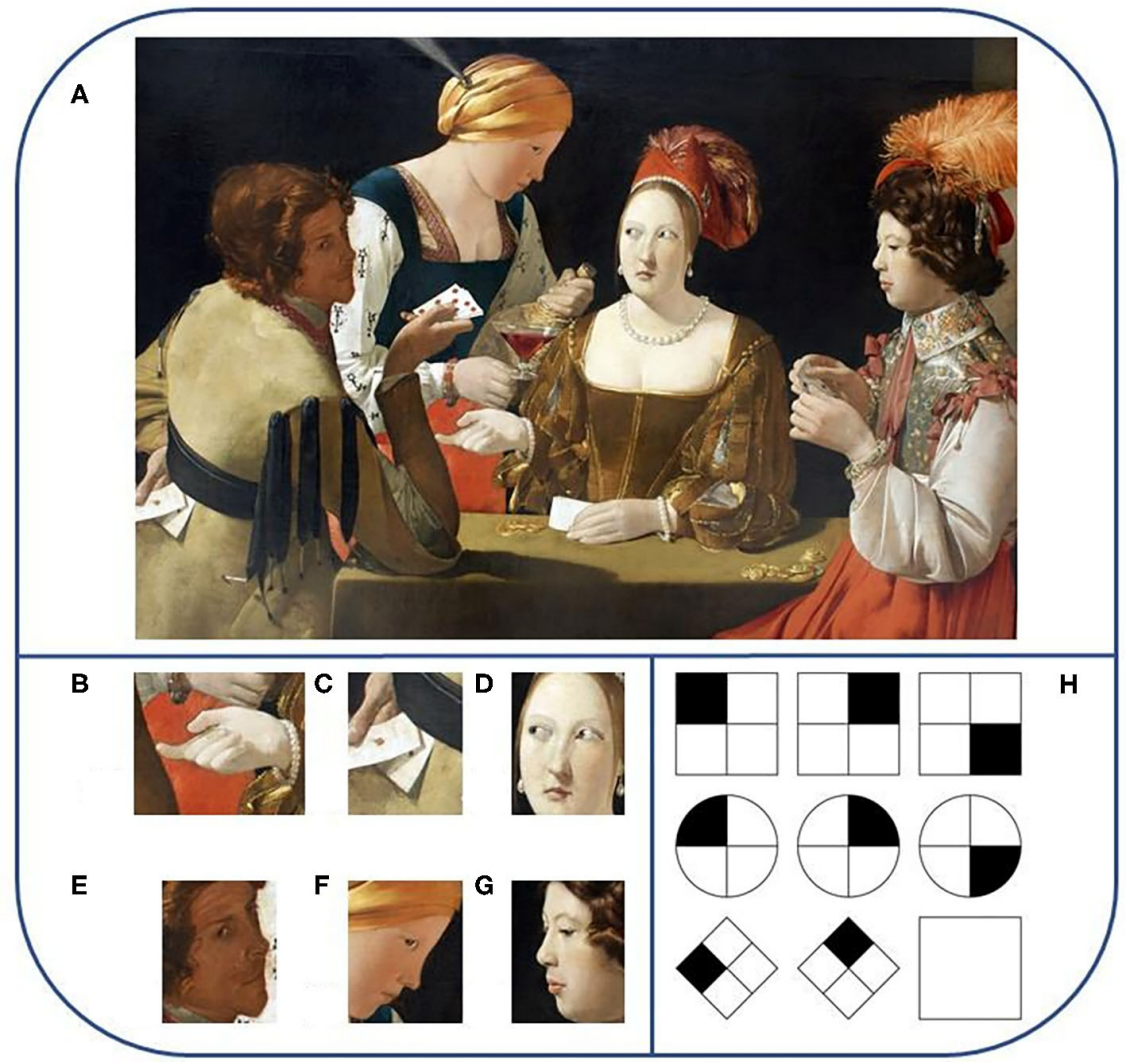
The difference between the complexities of social interactions and IQ tests. **(A)** The variables: we see a scenario of four people, their individual roles in the scenario, their relative positions in three dimensions to be estimated from a two-dimensional projection, occlusions of diverse objects and body parts for each person, the role of gaze directions (looking away, looking into the cards, looking at somebody), attention (seemingly no card-related attention, attention to own cards, attention to a person), objects in the hands (nothing, card or cards, glass of wine, bottle of wine), role of the hand (context restricted role of the hands within the context of the card game (showing the cards, hiding cards, covered pointing), the role of different cards in the game (ace vs. other cards), role of people (players, maid), the general posture and motion of the people (sitting, walking), and the related potential observations (unobservable and observable objects for different partners), hypotheses about past observation (has seen something), uncertain observations on facial expressions that the context can modify [compare **(A)** and **(B–G)** and the presumed goals (winning, making others to believe something, draw somebody's attention to something, asking somebody in case of uncertainty) as well as the irrelevant variables related to clothes, hair styles, jewelry, light intensities, amount of money on the table, including the varieties of forms, colors, shapes, and so on]. Reproduced from https://www.wikiart.org/en/georges-de-la-tour/the-cheat-with-the-ace-of-diamonds under Public Domain license (See also the cover page of Uta Frith's book: Autism: Explaining the Enigma, Blackwell Publishing, 1989). **(H)** A sample IQ test. The variables: (i) shape of the object (square and circle) is irrelevant, (ii) position of the black subfigure (clockwise rotation) is relevant. The subject is supposed to find the variables, to select the relevant one(s) and find the rule(s) between them. Reproduced from https://en.wikipedia.org/wiki/Intelligence_quotient under CC BY-SA 3.0 license. The search space for solving the problem and to come to the decision scales exponentially for both **(A,H)**. Searches may become exponentially more difficult as the number of variables increases, since the combination of the variables counts. Beyond that the number of variables is much larger for **(A)** then for **(H)**, probabilities, hypotheses are also involved in **(A)**.

Concerning the reduction of information flow, the number of variables required for success deserves special attention in a goal-oriented task. Problem solving tasks related to the social domain are more dynamic, complex, and abstract. Human behavior changes quickly due to our own actions, the actions of the partner(s), and the assumptions about the intentions of others toward us and vice versa, or toward third parties, or objects. These “parameters” are typically well-hidden from direct sensory observations, e.g., at the level of cones and rods. In addition, social interactions become more efficient when signals are provided, including utterances and gestures, tactile information that may also serve as means of deception (Figures 1A–G). Changes in these information sources typically need highly adaptive and very quick responses. The fast synchronization of actions in the distributed and relatively slow neural system is not trivial and may be corrupted in many ways and can be asserted further by errors of the internal reinforcing machineries.

By contrast, traditional tests measuring cognition in ASD are considerably limited to relatively simple multi-dimensional cognitive processes compensated by specific items related to social behaviors. In IQ tests, the dimensionality of the tasks is very small: mental manipulation of geometrical objects in matrix or verbal categorization tasks require the consideration of variables such as simple shapes, a few orientations, or colors, and only a few at a time. These tests are also *easy* in the sense that the objects of IQ tests do not interact or change in time which further simplifies the complexity of the cognitive tasks (Figure 1H). In addition, social interaction related factors have no influence on the performance.

The paper is organized as follows. In the next section, we review relevant information theoretical concepts and introduce our model built on autoencoding principles aiming at the learning of components. In Marr's terminology (3) these concepts are on the algorithmic level, but we also show “what” is computed and “why.” In the next section, we consider local and global neuronal features (implementation level) of autistic individuals that can (a) counteract the reduction of dimensions by means of component separation with (b) special emphasis on components relevant in the social context, (c) the encoding of the found components, and (d) their exploitation. Armed by these concepts and the experimental findings, we describe the autism palette, i.e., the different causes, or “colors” that are relatively small corruptions of processes in (a) to (d) *but* can lead to an ASD phenotype when combined. Impairments (section Impairments) lists examples of damaged implementation, while section Comparing Our Autism Palette Proposal With Other Theories of Autism helps to place the Autism Palette framework among previous unifiable theories of autism. Genetics (section Genetics) supports our hypothesis and suggests experiments for the dissociability of specific impairments. We review and combine our arguments in the last section.

## Parts of the Framework

This section reviews concepts concerning deep artificial neural networks, which serve us for modeling cognition. These concepts are selected with the aim to be used for the construction of a cognition-based unifying framework of autism, The Autism Palette. We start with *autoencoders* since we consider it as the core concept in modeling cognition. Autoencoders can develop compressed predictive models of the world serving the *making sense process* of internal and external sensory observations. During this process components are formed, stored, adapted, manipulated, and used. In the second part of this section the concept of components will be introduced and we elaborate on component types by means of Cartesian Factors (CFs). Later in this section specific parts of the framework will be discussed: emotional signals are treated as specific CFs that serve social communication, the complex nature of social interactions is considered, and we review the reinforcing means that help to solve social tasks, too.

### The Approach

We illustrate our concepts with the help of a few figures, by means of references to relevant computational results in the literature and via complexity considerations. We are constrained to follow this route for the following reasons:

1. Deep learning architectures that solve e.g., object recognition problems, video tracking tasks, performing pattern completion and alike require huge memories (on the order of 10 Gigabytes) as well as considerable training time. Beyond the training time requirements, the listed tasks are *relatively simple* and they need enormous databases. A complex architecture that could demonstrate autism related phenomena is beyond reach from the point of view of architectural, database, and training time requirements. We are restricted to computational principles and to certain relatively *simple features*.
2. A complex architecture has a large number of hyper parameters that are hard to justify one-by-one and the architecture can show colorful behaviors, while not being a faithful model for autism.

In turn, concepts in the paper will be presented by verbal descriptions and these verbal descriptions will be accompanied by references to the relevant and illuminating computational experiments in the literature, including some efforts of one of the authors (AL) and his group. These papers and the computational experiments may help the interested reader to find more details about the mathematical formalisms and the state-of-the-art results. Detailed thoughts and references to theoretical works can be found in the Supplementary Materials, and to some extent in the Figures of this section.

### Autoencoder

**Figure 2:**
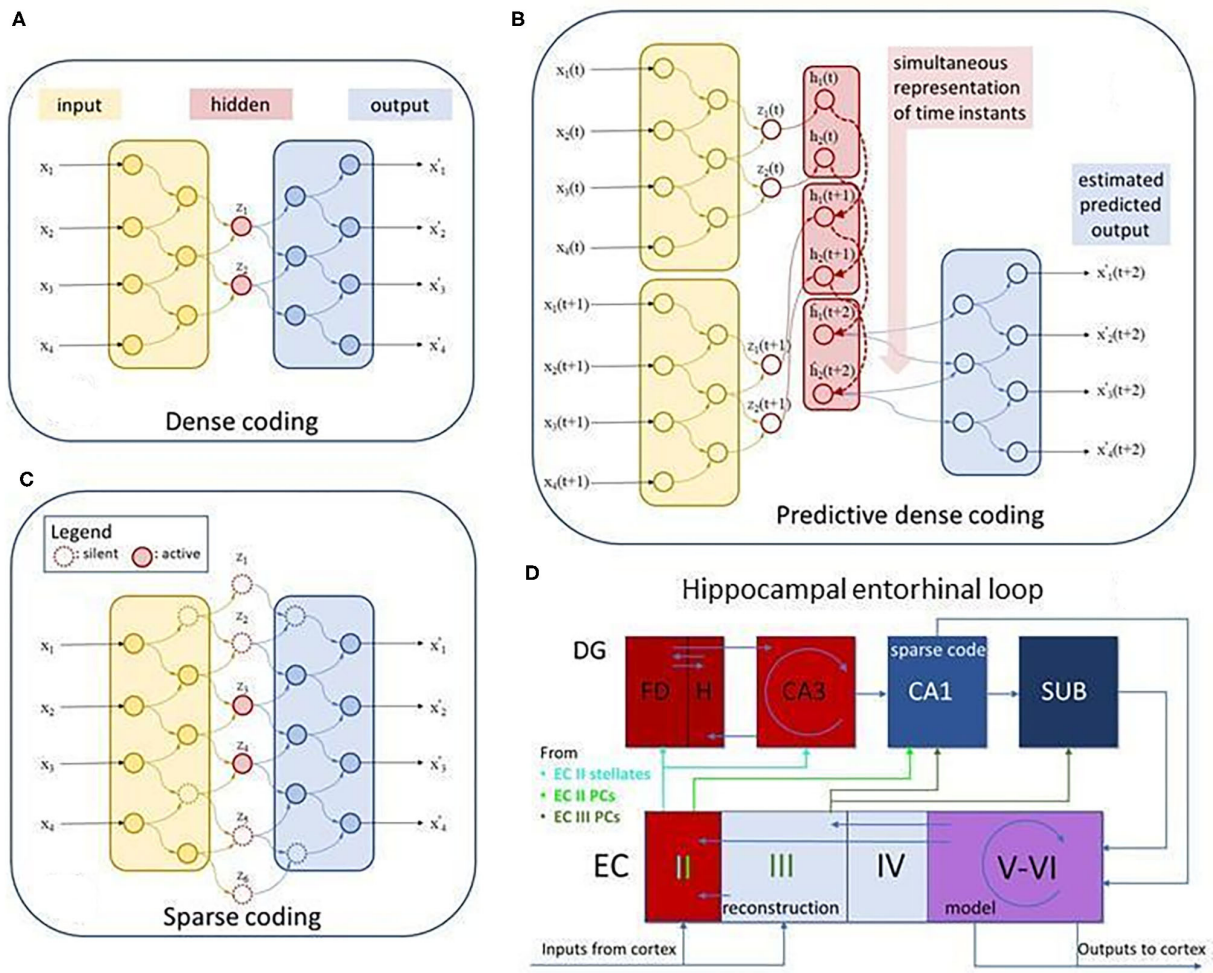
Dense, sparse, predictive dense autoencoders, and the putative autoencoding structure of the entorhinal-hippocampal complex. **(A)** Dense autoencoder. x: input, z: compressed hidden representation, or code, x-to-z mapping is the *encoding process*, x′: estimated input, z-to-x′ mapping is the *decoding process*, **(B)** predictive dense coding: h: compressed predictive hidden representation. **(C)** Sparse autoencoder. Same as **(A)**, but not all units are active at a time. **(D)** Hippocampal-entorhinal complex: FD, fascia dentate; H, hilus; DG, dentate gyrus; CA1 and CA3, CA1 and CA3 subfields; SUB, subiculum; EC II-III and EC V-VI, superficial and deep layers of the entorhinal cortex, respectively. EC II, putative error layer (e = x–x′); EC III, putative layer for input estimation (x′); EC V-VI, putative predictive model layers (h); Putative role of DG, detecting delays and learning to compensate delays; PC, pyramidal cell. Recurrent connections make a spatio-temporal model from the spatial one, propagate the representation forward in time for upstream estimation of the downstream signal and to compare them to produce errors for correction and for learning. See also Lorincz and Buzsáki (4), Chrobak et al. (5), and Lorincz (6).

Brains can imagine and dream. They can generate experience like vivid imaginations by manipulating their internal neuronal codes. The process is called generative process, or decoding process, which forms the decoding (also called confusingly top-down in AI, or downstream in neuroscience) arm of an autoencoder. The autoencoder (Figure 2A) is a computational architecture that has another mapping called bottom-up (encoding, upstream) arm.

Encoding maps the input to the so-called internal representation: encoding converts sensory neuronal responses to deep neuronal responses. Decoding uses those deep neuronal responses, the internal representation, and produces an estimation of the input. Encoding, decoding and in turn, the internal representation itself is learned. The guiding principle is compression as dictated by the need for component formation. Compression may come in a dense form and as sparse coding that will be described later.

The learning trick of the autoencoder is the matching of its input to its generated input, while compressing its representation. It follows from the considerable upstream and downstream processing delays that prediction is involved in order to match the actual input to the decoding of the old encoded one (Figure 2B). Delays, however, call for predictive autoencoders, to be treated later.

Compressions have two distinct forms. One type is the so-called dense code. This case is like the zip code: components are interlinked, and no individual element of the code may make sense (Figure 2C). The other version is called sparse code. In case of this type of compression individual elements of the code may represent individual higher than second order correlations in the input, such as the edges of images (7), the higher order correlation of pixels. Sparse encoding produces a few indices of the representation, whereas decoding estimates the whole input as the (non-)linear sum of the individual elements. For example, the sparse code of a face contains a nose, a mouth, two eyes and other parts of the face, see e.g., Figure 8 in Makhzani and Frey (8). If the input is the close-up, then components related to eyes, noses, and mouths become activated, and components unrelated to face, such as arm, hand, leg, torso, objects in general are not activated; only a few of the indices represent any input and, in turn, the representation is “sparse” (Figure 2A right hand side). Furthermore, representations related to objects, such as houses, cars, cups, even paper clips (9), or geographical locations (10) may take negligible contribution if any in the decoding if the input is a face. In this case, encoding selects a few units of the decoding network, producing a very compact representation of the input. Alternatively, if the input is a landscape, then, e.g., the components of the face are not activated. Compression, i.e., the number of the active units that represent the input can be smaller in the sparse case than in the dense without losing more information. This is due to the much larger number of computational units in the former enabling different combinations to play a role for different inputs and bringing about some level of explainability as well as symbiotic holistic and component-wise recognition (see later).

### Components

In this subsection, we elaborate on the concept of components in order to introduce our concept of Cartesian Factors in learning. Components in machine learning are distinct, sometimes autonomous parts of a larger system that can be hidden, may belong to an agent, or to its environment. As an example, the agent is a component of the environment and both the agent and its social partners are made of components that all undergo temporal changes. Components can be connected, may interact, and their coupling may give rise to reversible and irreversible changes in components. A typical component can be decomposed into smaller ones. An example is the eye, a part of the face, a part of the body that is made of components, such as the iris, the retina and so on. Components reduce combinatorial explosions and lower the complexity of cognitive tasks. For a short review on concepts of sparse coding, e.g., the concepts of “dictionary” and its “words,” and on component-wise and holistic recognition, see the Supplementary Materials.

**Figure 3:**
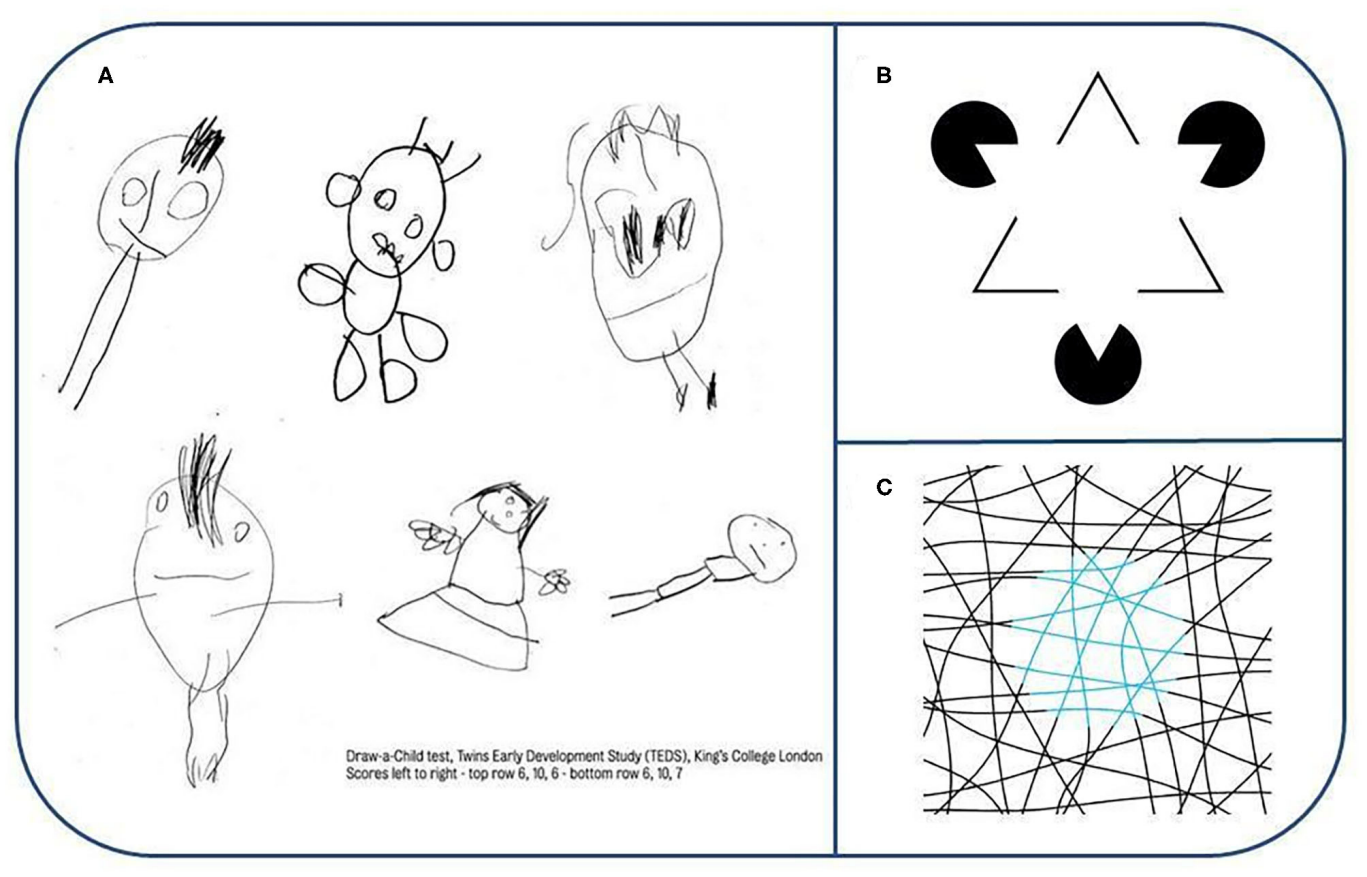
**(A)** Draw-a-Child test. Note the quality of the drawings and that many of the components of the body as well as components of the face are depicted in these drawings. Reproduced with permission from King's College London. Their work was supported by the following grants: MRC grant G0901245 & NIH HD044454 & HD059215. **(B)** Examples for component related illusion: Kanizsa triangle illusion. White color is identical everywhere. Edges—in our framework—are Lego-like, i.e., summable (CF1) components being inserted into the representation via a pattern completion machinery that eventually gives rise to apparent differences in color. Reproduced from https://en.wikipedia.org/wiki/Gaetano_Kanizsa#/media/File:Kanizsa_triangle.svg under CC BY 3.0 license. **(C)** Example for qualia related illusion: Neon color spreading illusion (lines are black and blue). There are only three colors—white, black and light blue—in the figure. Small rotation of the disk region that looks light blue removes the illusion, leaving only light blue lines against a white background (see at https://michaelbach.de/ot/col-neon/index.html). A pixel wise examination of the image reveals the true colors. The interpretation of the illusion is that color, a CF2, is separated from shape during processing and regions are refilled during the decoding process that may modify the input at certain levels of the processing hierarchy alike to the case of binocular rivalry (see text). Reproduced from https://commons.wikimedia.org/w/index.php?curid=29960445 under CC BY-SA 3.0 license. Original source: http://www.blelb.com/spot005/spot005_de.html.

**Figure 4:**
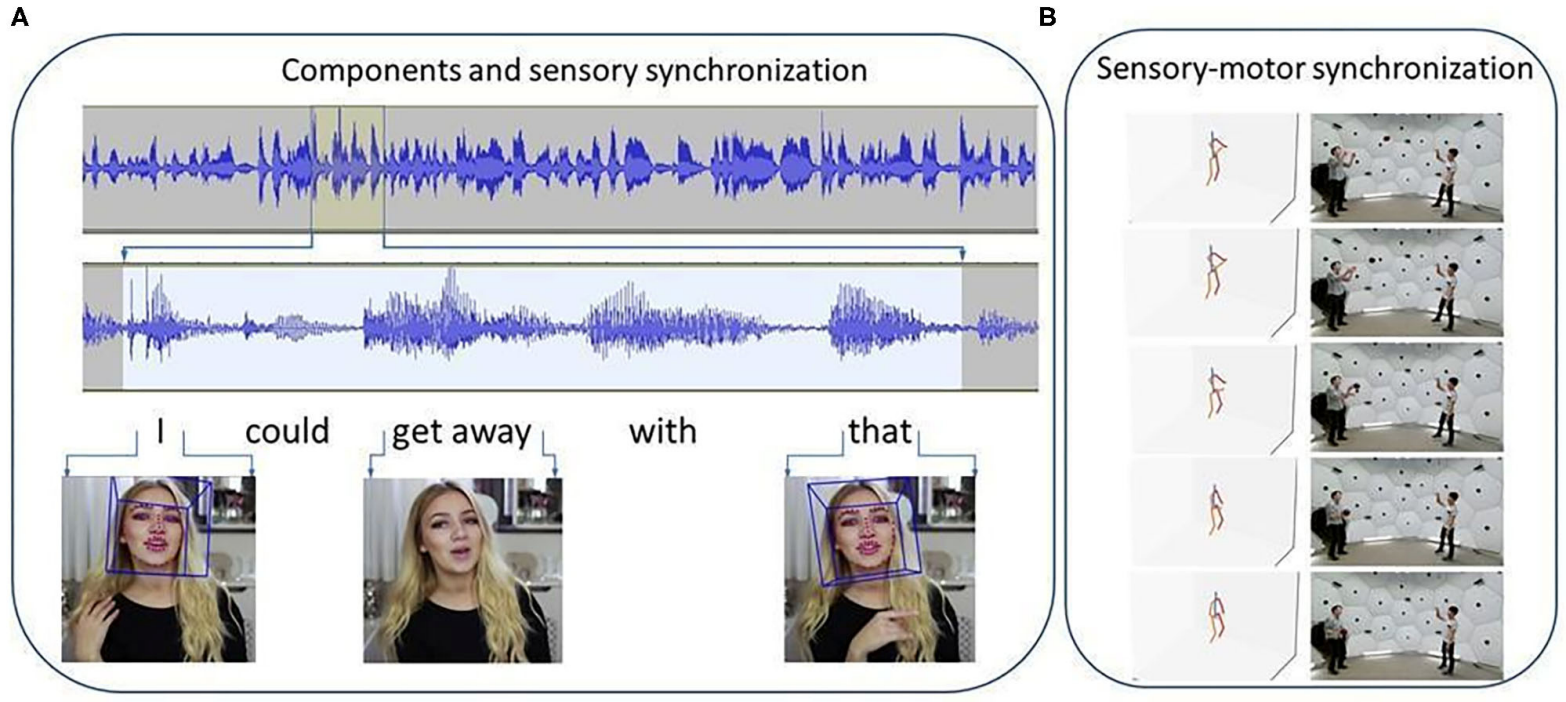
Components and synchronization problems at the multi-modal sensory level and at the sensory-motor level. **(A)** Delays exist between the visual and auditory modalities. Utterance: “I could get away with that.” Intense parts and more quiet regions follow each other In the acoustic domain, some of which are longer, others, like phoneme “t” in *that*, are so short (on the order of tenths of milliseconds) that they can't be seen in the enlarged part of the 15 s utterance. Separation of components concern, e.g., the separation of “get” and “away” that for a continuum. Components also include eye and gaze, hands, lip, head pose, hand pose, prosody, words of speech. Visual information is contained by the eyes (e.g., looking sideways at words “get away,” by the hand (e.g., pointing to the self and to sideways at words “I” and “that,” respectively). Visual and auditory delays are on the order of 100 ms (13). One hundred milliseconds shifts between auditory and visual modalities can be compensated well (14). Figures show our analysis using OpenFace (15) and Audacity^2^ software tools. Data is from the Chalearn database; code of movie: YJ5CINQ8v_E.005. **(B)** sensory-motor synchronization requires timed responses on the order of 10 ms or shorter. Illustration shows a deep learning-based estimation of a 3D body model from 2D camera input (16) in a video showing ball throwing from the Panoptic studio (17) database.

Components can be formed in diverse ways and psychologists suggested the Gestalt principles for learning the important ones a century ago (11). Children's drawings support the theory of sparse coding. Figure 3A shows a few examples from the “Draw-a-Child” test representing components of the body and components of body components, such as hand and fingers, eyes, and irises, among others. Similar principles have been used in computer vision [see e.g., (12)]. For a speech related example, see Figure 4A.

Our notion of CFs to be described below is, however, more general. Components, like nose, mouth and eyes, make the face when summed up. In many cases CFs do not sum up but restrict space and time: they can be product-like. The joint set of summable and product-like components (that together we call CFs) seem to provide explanations for a number of ASD impairments and for social interactions, in particular.

### Cartesian Factors (CFs)

There are many different formulations for “factors” and “components” in the literature, such as (1) non-negative components, (2) principal components, (3) independent components, among others, including their sparse multi-level (18), and (4) non-linear extensions (19). In order to distinguish our concept from these, we introduce the concept of CFs that come in two different forms. First, general CFs will be treated. Then, we turn to social behavior specific CFs.

#### The Concept of Cartesian Factors

Type 1 CFs, or CF1s for short are akin to traditional (summable) components. The main feature is that CF1s are decomposed from larger systems. For example, the nose, the eyes belong to the face and the face is a component of the body. CF1s are like Lego elements; they can be combined.

Type 2 CF (CF2) differ. A few examples for CF2 are colors, three-dimensional space, one-dimensional time, one-dimensional space of numbers and few-dimensional space of letters, or faces, as shapes.

Type 2 CFs are special in the sense that they do not exist by themselves. They can be modified but cannot be separated. They concern the type of the components under consideration. For example, any color is always bound to something. This is not the case for objects or episodes, since they occupy a local region in space and time, i.e., they exist *somewhere* and *only for a while* being parts of concurrent processes. By contrast, CF2s (e.g., color) are not limited either by space or in time. A somewhat similar concept in philosophy is called qualia [see e.g., (20) and the Stanford Encyclopedia of Philosophy]. Typical examples for qualia are the blueness of the sky and the scent of a flower (21), and (22), respectively. See the Supplementary Materials (a) for further characterization of CF2s and their relation to illusions and (b) for the AI approaches toward Type 2 CFs. Two examples on illusions are depicted on Figures 3B,C.

**Figure 5:**
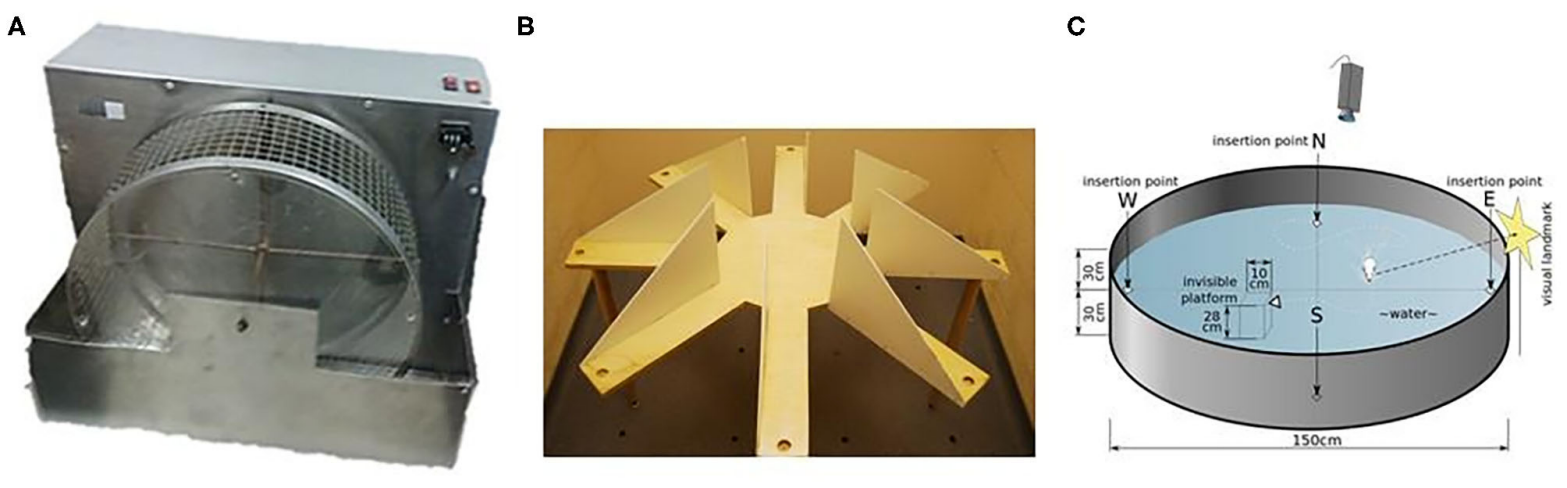
Point like, 1D-like and 2D-like mazes for rodent experiments. Hippocampal place cells follow the point like, 1D-like and 2D structure of the available space. **(A)** Running wheel. Reproduced from https://peerj.com/articles/2976/ under CC BY-SA 4.0 license. **(B)** Radial arm maze. Reproduced from https://en.wikipedia.org/wiki/Radial_arm_maze#/media/File:Simple_Radial_Maze under CC BY-SA 3.0 license. **(C)** Morris water maze. Reproduced from https://en.wikipedia.org/wiki/File:MorrisWaterMaze.svg under CC BY-SA 3.0 license.

As a specific example that may take the reader closer to our concept of CF2, consider mazes in different rat experiments (Figure 5). The constrained bottom region in a vertical running wheel (Figure 5A) is “point-like.” The radial arm maze (Figure 5B) is composed of a set of straight one-dimensional (1D) line segments, whereas the Morris water maze (Figure 5C) represents a two-dimensional (2D) space. On the other hand, bats live in a three-dimensional (3D) space (23). Place cells in the hippocampus are in the CA1 and CA3 subfields (Figure 2D) and signal in different local regions of the 1D-3D spaces of these animals (10). In turn, the set of place cells represent space independently from other details of the environment, so these sets are Type 2 CFs. At the same time, individual place cells divide space into elements of Type 1 CFs and each space region can be occupied or freed by obstacles.

In spite of the large differences between mammalian species, some of the CFs, like 2D or 3D spaces and the belonging metric develop similarly in rats, bats, non-human primates, and humans [see e.g., (23)]. Although the algorithmic and representational details of CF1 and CF2 formation remain unclear, many, if not all of them fall under the category of declarative memory, where the entorhinal-hippocampal complex plays a critical role during learning. We shall return to this point later. We finally note that CF2s may depend on culture and on scientific advances as demonstrated by (24).

#### Emotion as a Cartesian Factor

There are many components that need to be considered in social tasks; social tasks are complex (Figure 1A). Components include CF1s, like the individuals, who are present, absent, or occluded and may change quickly in social situations. Individuals can have complex interactions depending on their motivations, goals, and moods and these variables belong to the CF2 category. Such behavior-related factors are analogous to color, shape, and dimensions, but they are well-hidden. Emotion-related observations and interpretations can differ from individual to individual and may depend on culture.

The visible signs of emotions are multifaceted and include eye movements, blinking, hand gestures, body language, verbal expressions and alike, and the range of any given emotion can vary from person to person. The duration of emotions can span over shorter and longer time periods, with some emotion cues occurring in a flash. Combinations of visible gestures may change from time to time and may be the subject to self-control.

The “emotional space” of an individual may be approximated in two dimensions, see the “emotion wheel” of Plutchik (25) that, however, does not cover the related dynamics. There are many such deeply hidden factors and they may mix making social interaction dependent tasks hard to accomplish.

### Complexity of Behavior and Social Learning

Figure 1 depicts the differences between factor searches in IQ tests and factors that influence social learning, including the time constraints.

We recall the arguments that

a) Cartesian Factor formation is crucial for social interactions,
b) the quality of social interactions can be influenced by many internal behavioral facets, including motivations, fear, efficiency of reinforcements, among others,
c) the number of factors in social interactions can be very high (Figure 1A).

In addition,

d) learning of social interactions relies on social and non-social reinforcements as mentioned before.

In turn, learning is hard due to the large number of the involved factors and the additional burden of the temporal credit assignment problem that determines the relevant components that led to failure or success (26). For more on this subject, see the section on reinforcement learning in the Supplementary Materials. Note that (a) reinforcers can be either external or internal, including the social domain and (b) their nature can be direct e.g., food or money and indirect or social, such as the smile or the anger of another person.

Components are useful for (a) pattern completion by adding missing CF1s and similarly, one can consider prediction as pattern completion in time, (b) generalization via eliminating many details, including some of the CF2s, and (c) selecting only a few of the many CFs for decision making.

In the brain, many details of CF formation have been uncovered at least for the representation of space (10) and there are many theories on how CFs are formed, see the next section.

### Predictive Autoencoders

Learning of spatio-temporal components is sometimes supported by evolutionary means, especially if they are both complex and relevant, take the examples of treadmill stepping in infants (27), or the production of basic facial expressions of emotions (28): partial genetic basis seems to exist for these.

Learning of such components is hard due to processing delays that should be counteracted in such a way that sensory observations and conscious perception are to be synchronized for timely decision making. Synchronization with ongoing internal computations concerns *concurrent episodic components* including (a) decision making, which launches actions that may occur with a 100–200 ms delays and (b) the sensory signals about the launched actions that can be delayed by an additional 100–200 ms.

**Figure 6:**
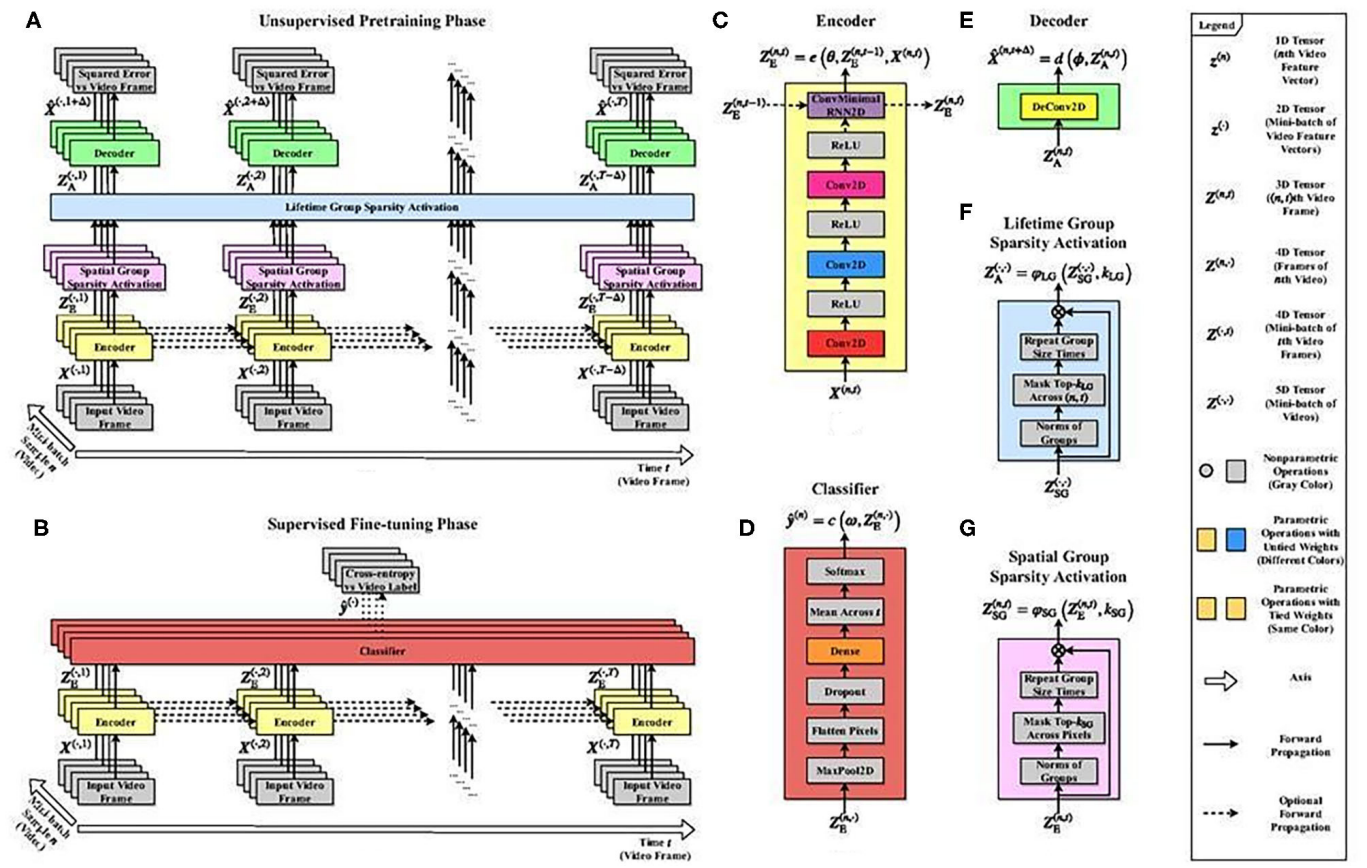
Illustrative predictive structured sparse architecture that models columnar organization. Schematic diagram of a neural network architecture with (i) many layers, (ii) recurrent extension of (iii) structured sparsity for modeling columnar organization. The architecture that reaches state-of-the-art performance (29, 30) illustrates architectural complexity for modeling a few of the autoencoding aspects of autism. Unmodeled features include, e.g., (iv) delay compensation, (v) different modalities, (vi) information fusion, (vii) synchronization between modalities, and (viii) the selection of a single percept. The number of parameters is still very large and offers too much freedom for modeling, while real data is uncertain. For example, autism-like features could be produced when modeling the contradictory findings on the distance and thus the speed and strength of interaction between minicolumns (31, 32). “Spatial group sparsity” sparsifies representation. “Lifetime group sparsity” keeps all units to play a role during the learning phases. Notation: subscripts E, A, SG, and LG denote encoding, activation, spatial group sparsity, and lifetime group sparsity, respectively. For more details, see legend. Details of the architecture: **(A)** The overall architecture is a deep convolutional neural network that takes *N* videos of *T-*1 frames as input and generates the video frame approximations forward in time in unsupervised pre-training phase. **(B)** In supervised fine-tuning phase, the videos are mapped to the video labels. **(C)** The encoder *e* is a deep, recurrent convolutional neural network that takes the input and produces the encoding, i.e., the hidden representation. **(D)** The classifier *c* is a dense neural network that takes the encoded hidden representations as input and computes the video class label approximation. **(E)** The decoder *d* is a single linear deconvolutional layer that takes the group sparse hidden representation as input and returns the video frame approximation. **(F)** The lifetime group sparsity activation function takes the spatial group sparse hidden representations as input and yields the lifetime group sparse hidden representations by keeping the feature map groups across sample indices and replacing the rest with zero. **(G)** The spatial group sparsity activation function takes the encoded hidden representation as input and calculates the spatial group sparse hidden representation by keeping the feature map groups across pixel indices and replacing the rest with zero.

An illustrative architecture having the key elements for compensating delays (29, 30) is depicted in Figure 6. It features a modular structure, a model of the minicolumnar organization that may play a role in autism (31, 32). For more details, see the Supplementary Materials.

### Summary of Section Parts of the Framework

In this section we introduced CFs, a special form of components. We argued that emotions can be CFs, too. We also described the concept of the autoencoder and its specific form, the predictive autoencoder. Social learning also requires high quality recognition of the social rewards and the proper evaluation of the reinforcers. Corruption in any of these elements should slow down learning and the individual may quickly accumulate negative rewards due to inappropriate behavior.

The main limitation of our approach is its verbal nature. Each of the concepts from this section have strong computational ground even the most complex ones, as shown on Figure 6. However, the Autism Palette itself is a verbal framework, which is not a “quantitative brain simulation.” In the following sections we will look at findings coming from neuroscience (sections Vulnerabilities and Impairments), from previous cognitive models (section Comparing Our Autism Palette Proposal With Other Theories of Autism), and from genetics (section Genetics) that support our framework.

## Vulnerabilities

In this section, we discuss some local and global features of the neuronal system that can contribute to our framework. They show many facets that serve dimension reduction of diverse kinds. Corruptions in the formation and manipulations of these features form potential vulnerabilities and make the colors that can be combined on The Autism Palette.

### Cartesian Factors in Different Brain Areas and Mammalian Species

The two types of CFs can develop in different ways as shown by computational models. Summable, i.e., Lego-like CFs (CF1s) seem to be present already in the primary visual cortex and they form a low-level dictionary (section Components, in the Supplementary Materials).

Sparse representation occurs at different sparsity levels in the brain. Consider the so-called grandmother cell (33). This theoretical cell that responds vigorously for any sensory information concerning the grandmother of someone combines a number of CFs (cloth, shape, motion dynamics, name, prosody, etc.) and thus the response to the input is extremely sparse (compressed). Similarly, neurons like the ones representing celebrities, e.g., Halle Berry neurons—that respond to her photos from different views, with or without sunglasses, dressed typically or in the Catwoman-dress, shown in line drawing, and by the letters of her name, while having negligible responses to anything else—also belong to the grandmother cell category (34). Grandmother cell like representations seem to form the words of a dictionary while they are still sparse and thus, summable.

In the primary visual cortex, the so-called simple cells respond optimally to moving rectangular bars of specific orientation, i.e., to the specific higher order correlations (35) of natural scenes (36). Such correlations, unlike random noise, are relatively frequent in natural scenes and thus responses are not too sparse, but they add up to segment objects and are like the words of a dictionary.

Learning of modifiable CFs (CF2s) seem to occur e.g., in the loop of the entorhinal-hippocampal complex (EHC loop) for representing space and the related metric that appear to be encoded in the medial entorhinal cortex [see the book of (37)]. The space related code has place cells firing in local regions of 2D in rodents (38) and 3D in bats (39), whereas metric related neurons fire along triangular grids in rodents (10). Both grid cell and place cell firings correspond to sparse population coding (40)^3^. Separation of the representation of space from everything else makes a CF2 system. Place cells themselves seem to form the words of a dictionary, i.e., they are CF1 components of the CF2 representation. This is similar to the separation of colors from other features and the division of the color space itself.

We summarize the properties of the sparse representation:

a) Cells respond sparsely.
b) They respond to higher order correlations and resemble to the words of dictionaries.
c) The level of sparsity differs in different areas
d) Responses are strong for diverse representations of the entity.
e) Type 2 CFs can be divided into CF1 subsets

In turn, simple cells, “grandmother cell-like neurons,” and place cells respond to different non-linear subspaces of the sensory input space and (a) the subspace has many details about the entity represented by the cell, (b) a small portion of the subspace is sufficient for the cell to provide considerable output and this response is highly specific to that entity. The same input is enough for the subject to recall other parts embraced by the subspace (33) suggesting that pattern completion ability is another specific feature of these cells.

### “Mirror Neurons”

Certain neurons in primate brains respond, e.g., to hands no matter if the hand belongs to the observer or not (41, 42). Such neurons have been termed “mirror neurons.”

“Mirror neurons” may emerge in a component extracting and learning system, since hands, alike to other body parts are decomposable, so they are Type 1 CFs. In particular, compression is better if a specific manipulation can be remembered and recalled independently from who, where and when did it and can be associated to the person if needed. In our view, “mirror neurons” are not mirroring anything. Instead these neurons represent components of the body and generalize over the owner. Component extraction, learning, and manipulation offers a reasonable explanation for the neurons that seem to be “mirror neurons.” Impaired component formation may give rise to impairments in this apparent behavior. Such impairments have been found (43), see more details later.

### Brainwaves

As mentioned before, the EHC loop is a key component of learning of episodic and semantic memories. Brainwaves play an important role in these processes. Buzsáki (44) suggested that during exploration, i.e., during the theta waves, neocortical information enters the hippocampus and induces weak and transient heterosynaptic potentiation in the CA3 subfield. Later, these weakly potentiated neurons and neuronal chains cause sharp waves (SPW) during consummatory behaviors that give rise to long-term synaptic modifications in the hippocampus. Long-term encoding of these memories involves complex patterns of brainwaves.

According to the experiments, SPW and slow-wave sleep (SWS) play roles in the consolidation and synchronization processes. SWS seems to be more involved than REM sleep in the consolidation process, but both types of sleep contribute (45, 46). Different brainwave frequencies can couple, and slow waves seem to restrict the time interval for coupling when faster oscillation occurs within slower ones, a phenomenon called phase biasing (47). In turn, slow oscillations can coordinate local processes globally, making SPW ripples a highly synchronous wave pattern in the brain. Boyce et al. (48) showed causal evidence that contextual memory consolidation depends also on theta rhythms during REM sleep.

Brainwave machinery is highly specific over about four orders of magnitude in the frequency domain from 0.05 Hz to about 500 Hz (Figure 1A in Buzsáki et al., 2003). The linear progression of the different frequencies on the logarithmic scale is robust against brain size from mice to elephants. Errors of the brainwave machinery may have strong effects on the forming and the synchronization of the component-based memory system.

We turn to the autoencoder model inspired by the features of space encoding and the related metric in the EHC loop.

### Autoencoders in the Brain

The predictive generative nature of neuronal encoding, i.e., that encoding is accompanied by predictive decoding forming an autoencoder has gained considerable support over the years [see e.g., (49)]. It is customary to say that an autoencoder is “dreaming” when decoding is driven by the freely running predictive networks (Figure 2B) without any input.

It has been suggested that the EHC loop forms a predictive autoencoder (4, 5) that (i) can learn from errors of the estimations via the two-stage operation, (ii) can learn and compensate for delays, and (iii) can develop independent components. This line of research has been progressing; metric learning, learning of episodic representations (50), and semi-supervised learning of *sparse* Cartesian Factors (51) have been included. The architecture of the EHC loop is depicted and detailed in Figure 2D. Findings on learning to compensate for delays in the hippocampus (52) reinforced the model.

### Consolidation of Emotional Components

Emotional conditioning has been dissociated from declarative memory by Bechara et al. (53). Based on their results the amygdala is necessary for emotional conditioning, while the hippocampus is required for forming declarative memories.

The amygdala theory of autism appeared early (54): the amygdala was proposed to be one of several neural regions that are abnormal in autism. The theory is based on the general agreement that emotions are processed by the amygdala and it is supported by the anatomy:

The ventral hippocampus projects directly to the basolateral amygdala and the central amygdala, and connections are reciprocal (55),

a) fear can be switched on and off in distinct circuits between the amygdala, hippocampus, and the medial prefrontal cortex by selective activation of specific neuronal circuits (56), and
b) multiple parallel pathways exist between the amygdala and the hippocampus. One pathway encodes the context-dependent retrieval of cued fear memories. Another pathway is concerned with fear behavior in a context dependent manner (57).

In turn, fear is an internal component that may modulate both episodic and semantic memories. Experimental studies on the consolidation of emotional components have been published (58). They found that reactivations of memory traces in the basolateral amygdala peaked during hippocampal SPW ripples, being in line with other consolidation patterns.

### Neurotransmitters in Social Behavior and Reward

Component formation requires reinforcement learning based selection to find the relevant components, i.e., to solve the credit assignment problem in time (section Complexity of Behavior and Social Learning).

#### Dopaminergic Pathway

**Figure 7:**
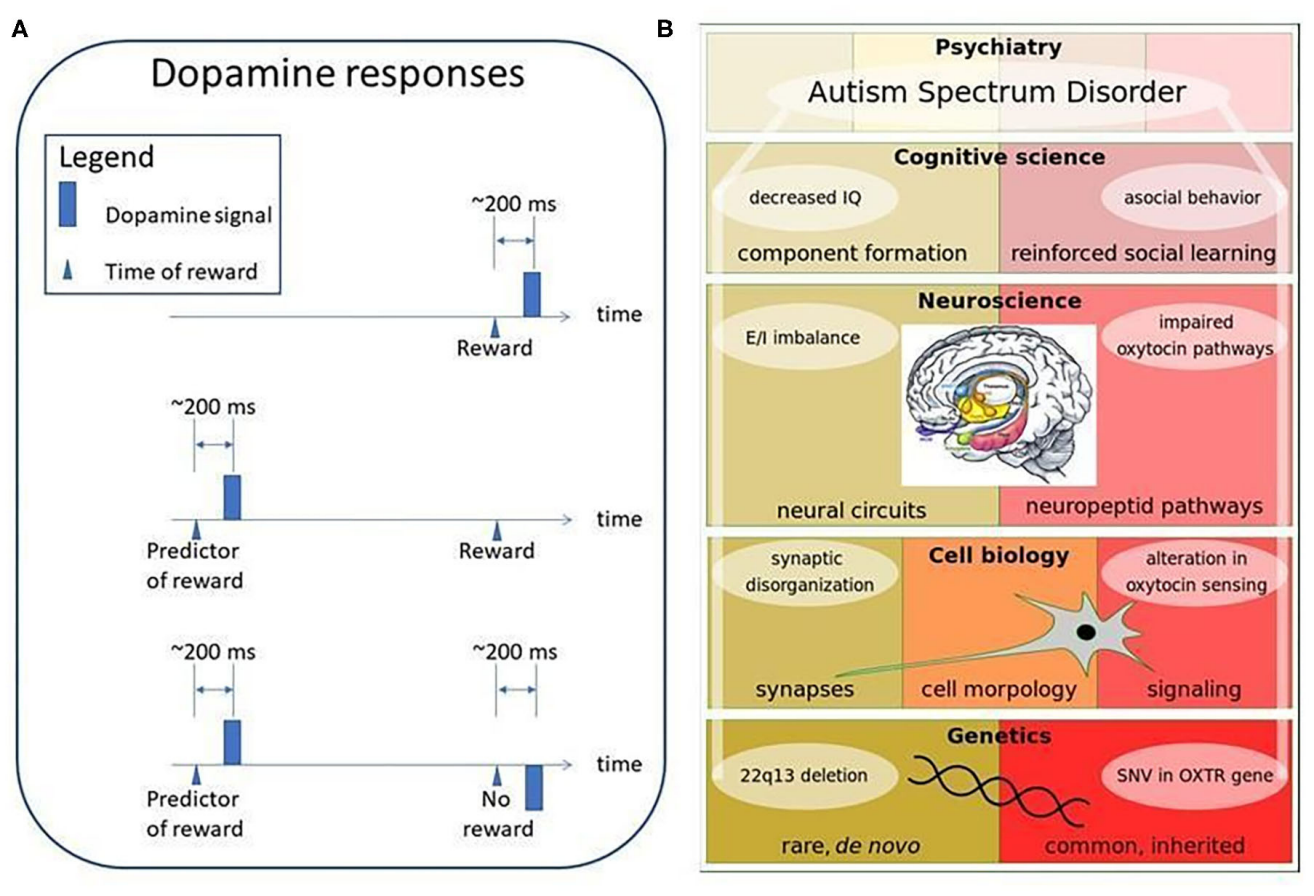
Dopamine responses and ASD stratification possibilities. **(A)** Dopamine responses. Dopamine signal emerges for reward, first. If the reward predicting feature is learned, then dopamine signals only for the predictive feature upon learning. If a predictive signal is present, but the reward is missing, then a negative dopamine signal appears at the time of the reward (60, 62). Mechanism in the striatal region is the same for processing either social or non-social rewards being aberrant in ASD individuals (63). **(B)** Examples of ASD stratification possibilities. Causes of ASD can be separated on different levels of biology (genetics, cell biology, neuroscience, cognitive science). These levels are built on each other and form causative pathways. Two examples are shown: (left hand side): the component formation related Phelan-McDermid syndrome (64), which is defined by a *de novo* 22q13 deletion as the genetic cause and synaptic disorganization, E/I imbalance and decreased IQ as consequences; (right hand side): the reinforced social learning related oxytocin system, where common, inherited SNVs in the OXTR gene could be a genetic cause and altered oxytocin sensing, impairments of the oxytocin pathway and asocial behavior as the consequences. Inset is from Sokolowski and Corbin (65) under a CC license. Color notation in the inset is as follows: amygdala is green, bed nucleus of stria terminalis (BNST) is light blue, hypothalamus (Hypo) is yellow, hippocampus (Hipp) is pink, mammillary bodies are orange, olfactory bulbs (MOB) is purple, amygdala is green, gray smaller regions are nucleus accumbens (NuAc), periaqueductal (PAG), and ventral tegmental area (VTA).

It has been known for almost 30 years that the dopaminergic pathway plays a role in reinforcement learning (59–61): the brain seems to perform temporal difference learning (TD-learning) depicted in Figure 7A. TD learning fires for unexpected rewards. As soon as the reward is well-predicted, it fires at the appearance of the predictor, but does not fire for the reward itself. Model based prediction and predictive autoencoders seem to be involved here. Pathway impairments will spoil the optimization of behavior and, in particular, social learning. Recent work indicates that reward processing is atypical in autistic individuals (63).

Beyond impairments in social learning, this pathway influences the learning of motor control. For a theoretical description and the review of the neuronal findings [see e.g., (6, 66)] and the references therein. Experimental results also show the complexity of potential genetic influences: polymorphism in the DARPP-32 gene play a role in probabilistic reward learning, C957T polymorphism of the DRD2 gene associated with avoiding choices that give rise to negative outcomes stochastically, whereas the Val/Met polymorphism of the COMT gene is involved in the ability of rapid adoption from trial to trial (67). The contribution of this pathway in autism has been demonstrated in the literature [see e.g., (68, 69)], and the cited references.

#### Oxytocin

Oxytocin (OXT) can affect a network of circuits and can coordinate complex brain functions to form specific behavioral phenotypes (Figure 7B, right side). Function of OXT is intensively studied in social behavior and autism. From recent works a specific network of neural circuits can be concluded. OXT release from the hypothalamic paraventricular nucleus promotes prosocial behavior by increasing the excitability of the dopaminergic neurons in ventral tegmental area (VTA), which are projecting to the nucleus accumbens. This circuit is a positive, *rewarding loop for social interactions* (70). These projections are part of the mesocorticolimbic dopamine (DA) pathway, a network of circuits, which was shown to be impaired in ASD in multiple studies (71, 72). In contrast to the positive effect of OXT on DA neurons of VTA, OXT indirectly inhibits DA neurons in substantia nigra, hence suppressing exploratory locomotion (73, 74). Impairments in the substantia nigra were implicated as *cause of repetitive behavior* in ASD (75, 76). These results connect the above mentioned OXT and DA circuits to two of the core behavioral symptoms of ASD.

## Impairments

In this section, we list examples of ASD related causative findings and how they are connected to vulnerabilities and impairments in our unifying framework. We list these impairments and—in certain cases—mention certain possibilities that may promote therapeutic procedures. We also consider ASD comorbidities as a special mixture of impairments. Following our analogy, comorbidities are the mixed colors on the palette.

### Disturbed Neuronal Growth and Morphology

Piven et al. (77) studied high-risk siblings early in their first year and performed neuroimaging studies beyond their first year. Some of them were diagnosed with autism later. They found cortical area hyperexpansion in the first year. In the second year, brain volume overgrowth followed and seemed to be associated with the emergence of social deficits. The relevance of these findings is shown in (78): within high-risk siblings, information about brain surface area can predict a positive diagnosis of autism with predictive and sensitivity values of 81 and 88%, respectively.

Larger surface areas of the same volume involve different gyrification patterns, as have been reported in the literature for the left pre- and post-central gyrus (79). Increased gyrification seems to enable an increase in the number of short-distance connections reviewed by Courchesne and Pierce (80) and that may corrupt the separation of encoded components.

According to Buzsáki et al. (81), axon size and myelination seem to be the most important factors for the scaling of network oscillations because they determine the conduction velocity of neurons. The slower the conduction velocity, the larger the delay to be compensated by predictive models to be learned and, in turn, the slower (harder) the learning may become.

Ecker et al. (79) found that increased gyrification is accompanied by atypical neural axial sprouting, which is most pronounced in axons traveling close to the cortical sheet. They report that enhanced gyrification correlates with increased axial diffusivity in general, and the relationship may be causal in either direction. Increased axonal sprouting may give rise to erroneous associations and that may also increase irrelevant incoming information, i.e., the noise level and spoil the precise timing of brainwaves and thus predictive encoding, too.

There are shared genetic factors behind anatomical changes of the cortex and general cognition (see section mTOR Pathway in the Supplementary Materials). Also these factors are parts of key regulatory pathways, hence their therapeutic potential is limited (82).

### Noisy Brain and Excitation/Inhibition Ratio Imbalance

Synaptogenesis is an additional vital matter. Changes in spine morphology can be critical in forming large networks, and troubled synaptic connections are considered the main underlying reason for autism by many researchers (83, 84). According to the data, increased spine density gives rise to decreases in cognitive functioning in ASD, supporting the view that ASD can be characterized by denser connectivities locally and hypoconnectivity globally (85). Both connectivity changes can corrupt global and specific local information processing in the brain. We note that synaptic genes are among the major ASD candidate genes, see section Synaptic Impairments Can Disrupt ASD Related Circuits in the Supplementary Materials.

Learning is harder in noise since either the noise has to be filtered out that slows down the learning process, or the noise will also be learned that limits the generalization capabilities. Filtering, however, is to be learned and part of the relevant information may be eliminated if filtering is imprecise. At the level of cognition, noise may affect attention, may cause hyperactivity, may restrict behavioral repertoire to avoid corrupted behavior, may give rise to a high variability of responses and so on. The emerging behavior also depends on the environment and the internal reward system.

Rubenstein and Merzenich (86) suggested that ASD is the result of noise in the brain. Markram et al. (87) proposed that hyper-functionalities in reactivity, plasticity, perception, attention, and memory become debilitating in ASD, causing social and environmental withdrawal, and locking the individual into a small repertoire of proven routines. In other words, the brain is not noisy, but highly responsive, and strong influences are dampened by restricted behaviors. The investigators studied the valproic acid rat model; valproic acid is an anticonvulsant and mood stabilizing drug that has been used for epilepsy and schizophrenia. Based on their studies, they propose that the high excitation/inhibition ratio has behavioral consequences.

Davis and Plaisted-Grant (88) argue that ASD symptoms reflect too little instead of too much neural noise. They argue that (i) the stochastic resonance observed in single unit recordings is influenced by additive noise that may give rise to improved detection and discrimination thresholds; (ii) noise facilitates transitions between observations, but such transitions can be slow or even missing in binocular rivalry, as reported for ASD (89), a potential corruption of observation. Furthermore, (iii) for similar inputs belonging to the same category, generalization between the inputs may become easier if the noise level is higher, a well-known strategy used in non-linear denoising autoencoders (90). The opposite may also occur if irrelevant noise is learned: generalization processes seem inefficient in autism (91, 92).

David et al. (93) consider not the strengths but the variability of cortical oscillation patterns. They note that searches for abnormal power spectra provide inconsistent results in autism. They emphasize that trial-to-trial variability in cortical oscillations form operational noise in neuronal networks that should consistently communicate between remote areas, and such variations have been found in ASD.

Dickinson et al. (94) reviewed the literature on excitation-inhibition balance in ASD and found that imbalances are essential and there is supporting evidence for such changes. Individual variability seems relevant, since the evidence justifies neither a net increase in excitation nor a net increase in inhibition in autism on the average.

Recent studies of infants with ASD led to intriguing novel findings on cascades of network efficiencies (95). Network inefficiencies were found in infants at high risk of later ASD before symptom consolidation. Inefficiencies—detected by MRI seed-based tractography measures of connection length and strength—were first apparent in low-level sensory processing as early as the age of 6 months, but only in short-range cortico-cortical connectivity. Inefficiency then spread to higher-level processing, and ASD symptoms appeared. Symptom severity can be predicted by the inefficiencies measured much earlier in low-level processing. Lewis et al. suggest that children with ASD may suffer from diminished synaptic pruning during early development.

In sum, endogenous noise, and excitation-inhibition imbalance in the context of ASD are controversial, but seem to be present in different cohorts measured in different ways and with different extents of ASD symptoms (94).

Brainwave abnormalities should affect component forming as well as the cognitive manipulation of components. Consistently, there are EEG abnormalities in autism and the abnormalities correlate with associated phenotypic features (96).

There are promising therapeutic methods to alleviate the E/I imbalance and related impairments (97), hence their dissociation from impairments with limited therapeutic potential would be beneficial.

### Impairments of the Amygdala and the Oxytocin Pathway Are Leading to Altered Social Behavior

Recent studies show that in comparison with controls, amygdala volume is greater in ASD (98). It was also found that higher endogenous oxytocin levels correlate with weaker functional coupling between amygdala and hippocampal regions and with higher attachment scores in adults with ASD (99).

Impairments of oxytocin pathways were noted in multiple studies in mice. For example, abnormal social interaction can be triggered by VTA DA neuron specific deletion of OXT receptor (70) or *neuroligin-3* (100), an ASD-related synaptic adhesion molecule. Moreover, mimicking an ASD related loss-of-function mutation of *neuroligin-3* in rodents increased repetitive behavior and aggression, which could be reversed by Risperidone, a D2 DA antagonist (101). These receptors and adhesion proteins are coded by genes, affected by genetic variants: they are factors of molecular pathways and their deregulation can disrupt neuronal circuits that have importance in ASD related behavioral phenotypes. In turn, certain genetic variants can strengthen vulnerabilities related to oxytocin mediated social reward that can lead to specific behavioral changes (Figure 7B, right side).

Oxytocin treatment may have therapeutic value for patients with cognitive impairments in the social domain (102). In turn, uncovering that impairments are connected to the oxytocin pathway gene networks (103) may shed light on both the need and the possibility of specific treatment, underlying our approach toward the dissociation of impairments.

### Clinical Consequences: Comorbidities

Below, we consider a few comorbidities found in individuals having ASD. We propose that these comorbidities are combinations of certain impairments, hence they could be used for the dissociation of the colors of The Autism Palette.

#### Epilepsy

Epilepsy is a neurological condition. It is often comorbid with other neurologic and psychiatric disorders. Epilepsy shows clinical overlap with ASD and has many other comorbid profiles. The prevalences of epilepsy in autistic males and females are ~18% and 34%, respectively (104).

Epilepsy is considered a network problem (105). As such, it is fragile in many ways, including network centrality (106). Seizures may be due to high excitation/inhibition ratios, one of the main theoretical routes proposed in autism models (86). ASD and epileptic encephalopathy seem to have many common genetic causes (107).

Cognitive impairment is the most common outcome of epilepsy; epilepsy can cause considerable harm to the developing brain (108): epilepsy gives rise to morphological and physiological changes, modified synaptogenesis and altered excitatory and/or inhibitory balance, which destroy both network structure and dynamics and increase the severity of the component formation impairment.

Mesial temporal lobe is the cornerstone of component-formation and memory consolidation. In turn, it is a falsifying issue what happens in case of mesial temporal lobe epilepsy that corrupts this key structure. According to (109), such epilepsy gives rise to social cognitive deficits, including significant mentalizing deficits, supporting our theory. Furthermore, Gelinas et al. (110) found that spontaneous hippocampal interictal epileptiform discharges correlate with impaired memory consolidation offering a straightforward connection between epilepsy and the impairment of component formation and thus the comorbidity of ASD and epilepsy.

#### Schizophrenia

Definitions of ASD and schizophrenia have changed over time. ASD, which was first described by Kanner (105) and Asperger (111) used to be considered as an early version of schizophrenia (112) or a central feature of schizophrenia (113). The opinion that they are different disorders started later (114), since ASD starts during childhood and is characterized by deficits in social interaction and communication, whereas schizophrenia typically has a later onset and is characterized by psychotic symptoms.

In addition to behavioral phenotypes, there are genetic links between ASD and schizophrenia [see e.g., (115); and the references therein]. We list a few similarities and dissimilarities in binding and low complexity component formation in epilepsy, schizophrenia and in ASD since these may influence the learning of Cartesian Factors.

Binding of sensory information between different modalities helps the fusion of information and helps pattern completion when part of the fused information is missing. Problems with bindings have been observed both in schizophrenia and ASD, e.g., in pairing audio and visual signals and in binding interoceptive signals (116). Binding requires precise temporal windows, but experiments with ASD patients show expanded audiovisual temporal binding windows and completely diminished temporal acuity for perceiving cardiovisual (interoceptive to exteroceptive) information (116). However, it has been questioned, if corruption of binding information sources has the same causes in ASD and in schizophrenia (117).

Errors in binding give rise to errors in predictive model learning. Take self-tickling as an example. Insensitivity to self-tickling should not be surprising given a precise model of the self, and indeed, typical individuals cannot tickle themselves. This remains the case even if bodies are swapped in the body transfer illusion (118). However, self-produced touch results in more ticklish perceptions in individuals with Asperger's syndrome than in normal subjects (119), and self-tickling is particularly successful for individuals with pronounced schizotypal traits (120). Precise temporal binding may require predictive synchronization on the order of milliseconds in the presence of information time mismatches of a few hundred milliseconds due to different propagation times. Sensory-motor synchronization is crucial and motor-sensory communication can be troubled in schizophrenia (121). Two illustrative examples, one on auditory-visual and one on sensory-motor binding are shown in Figures 4A,B, respectively. Synchronization errors may cause problems in the separation and learning of the components, the putative task of the entorhinal-hippocampal complex. Furthermore, the temporally adjusted encoding of the information into diverse areas of the neocortex for the sake of future recalls is also demanding. In turn, both component encoding and component recall may be impaired in schizophrenia with the possibility of comorbidities with autism by impairing the complex, multimodal requirements of social tasks.

A more complex pattern appears for the so-called “rubber hand illusion” [see e.g., (122) and the references cited] and the fast malleability of body representations into the environment, see, e.g., Tsakiris et al. (123). Experimenting with such fast malleability, as in the rubber hand illusion, Noel et al. (117) found that patients with schizophrenia and ASD behave very differently. Schizophrenia patients had a weak or variable bodily boundary between the self and the environment, whereas ASD patients had a sharp boundary. In our framework the difference can originate from the low complexity of the separation of the self from the environment that corresponds to the separation of self-controlled components from the rest of the world and seems relatively simple among the problems of component learning, unless synchronization impairments counteract it. In turn, as mentioned before for the case of IQ tests, much less impairment is expected in low-complexity tasks compared with higher-complexity tasks for ASD patients. Furthermore, since learning is more focused on the self than on partners in ASD, learned boundaries may be more rigid for individuals with ASD.

#### Synesthesia

Baron-Cohen et al. (124) report that the incidence of synesthesia, in which a sensation in one modality involves perception in another one, is approximately three times higher in autistic adults than in normal subjects (125) and found that synesthesia in autism is linked to savant skills. These results are further supported by Ward et al. (126), who showed that synesthetes have enhanced perception and attention and exhibit autistic-like impairments, too. It has been found that axonal connections between V4, which is involved in color processing, and the so-called “grapheme area” are denser in synesthetes than in controls (127). Both of these areas are in the fusiform gyrus, and some portions can be adjacent. Other findings support a cross-activation model (128) between these areas, and some of these cross-activations seem to be preconscious. Cross activation due to denser axonal connections may be able to corrupt component encoding into distinct neocortical areas. In turn, the comorbidity of autism and synesthesia seems to arise from network effects, e.g., from increased axial diffusivity, which can be fostered by enhanced perception and attention to either colors or graphemes, among other similar phenomena that restrict the separation and the cognitive manipulation of components, the central assumption of our model.

## Comparing Our Autism Palette Proposal With Other Theories of Autism

There are many cognition-based models of autism supported by both information theory and experiments. There are also social oriented theories. In this section, we consider their merits from the point of view of our framework. We focus on the limitations of the previous theories and how our model can overcome those.

### Cognitive Impairments

Cantio et al. (129) studied cognitive-level symptoms and searched for a universal pattern of cognitive impairments in ASD. They found that two such impairments—(i) impaired theory of mind, i.e., theorizing about the hidden mental states of other people (130), and (ii) impaired executive function, manifested as repetitive and stereotyped behaviors, among other characteristics—predict autism at a rate of up to 75% (50% being a random association). Tests on embedded figures (131) showed that local processing bias has non-significant contribution with close to normal distributions of similar variances. Their results support the idea that the social behavior impairments in autistic individuals may arise from potentially different causes, which strengthens the idea of the dissociable impairments. Cognitive impairments, however, provide no explanation for the IQ distribution in ASD and, in particular, for the relatively high number of autistic individuals with above average IQ. Impaired social motivation with intact general cognition concerning low complexity IQ tasks could explain this paradox to some extent.

### Weak Central Coherence Theory

Weak Central Coherence (WCC) theory is an early insightful model for autism. Frith and Happé (132) put forth the idea that autistic behavior is the result of impairments in extracting global form and meaning. Later, the model was modified (133) to say that problems might arise from the superiority of local processing, which is a bias in the processing strategy, and weak coherence may not be the cause but a symptom of autistic behavior. Robertson and Baron-Cohen (134) object to the theory on the grounds that WCC is a top-down mechanism and that bias in the cognitive strategy can hardly explain low-level sensory processing features. However, our component-based model may resolve this contradiction: If top-down mechanisms are supported by distributed sparse representations in an autoencoder and if elements of the two types of Cartesian Factors are not well-formed, then central coherence can be weak due to the lack of the relevant CFs since not-yet-separated CFs corrupt rigorous inference. In addition, *making sense* in higher dimensions is exponentially harder.

### Bayesian Prior Theory

Pellicano and Burr (135) suggested the use of Bayesian models to understand autistic information processing. According to them, differences lie in the perceptual mechanisms; namely, people with ASD have “hypo-priors” that give rise to unique, highly precise perceptual experiences. Based on this assumption, Pellicano and Burr claim that many autistic characteristics, from sensory processing to non-social impairments stem from differences in Bayesian prediction processes.

A recent work by Palmer et al. (136) summarizes the proposal that in autism, sensory information has larger weighting than in normal people. They argue that the balance between perception and action may be the characteristic difference between people with autism and normal people—in both social and non-social behaviors.

There is little doubt that Bayesian inference is difficult to discount, and it seems that this strategy is applied by the brain [see e.g., (134, 137, 138) and the cited references therein]. On the other hand, the world—apart e.g., from partial observations—is close to deterministic and actions with deterministic outcomes are both possible and desired and the Bayesian account may be limited in this respect: Experimental findings on endogenously and exogenously modulated binocular rivalry (139) seem to contradict simple Bayesian principles, since a Bayesian observer would always pick the higher-probability interpretation. The switching phenomenon, i.e., that perception switches from one potential interpretation to the other, however, indicates that simple Bayesian models are incomplete. Still, the fact that binocular rivalry is slowed down in autism seems consistent with the Bayesian observer assumption.

The assumption of hypo-priors can be justified by specific genetic differences concerning e.g., the oxytocin pathway or the dopaminergic projections. Comorbidities, however, are hard to explain on the basis of the Bayesian model, while they are straightforward outcomes of the Autism Palette model.

### The Empathizing–Systemizing (E-S) Theory

**Figure 8:**
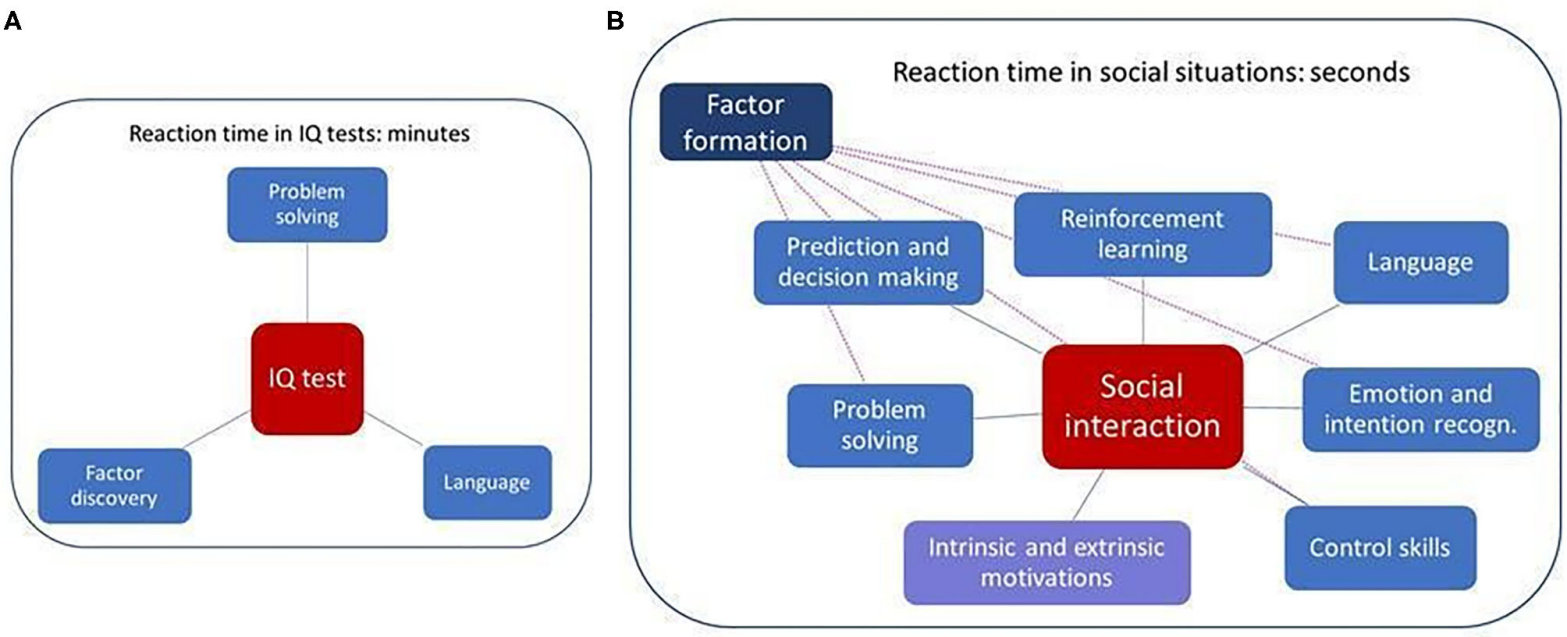
Time constraints and components needed for solving **(A)** IQ tests and **(B)** goal-oriented tasks in social interactions. Solid lines: main components, dashed lines: dependencies from the point of view of factor formation. Components of typical IQ tests are limited to Type 1 Cartesian Factors (CF1), including e.g., matrix tasks like in Figure 1H, or pattern completion task related to language. The number of components of social interactions is much larger and concerns both CF1s and CF2, involving a much larger search space of problem solving. Temporal constraints differ, too; time for an IQ test is prescribed and is on the scale of minutes, whereas social interaction may be much shorter (ms domain), but can be considerably longer as well. Differences are (a) in the complexity of the two problem types and (b) in their static (IQ) vs. dynamic (social) nature, too.

According to the E-S theory (140) individuals can be characterized on an empathizing and systemizing axis based on their social and non-social capabilities. Both empathy and systemizing can be the means for searching and finding hidden CFs. Affective empathy assumes proper emotional reactions (141) that could be corrupted. According to the E-S theory limitations in the social domain stem from the orientation being strongly directed to non-social tasks. This theory offers an explanation to ASD cases with superior non-social skills and above average IQ. The theory needs to be extended with complexity considerations, since social and non-social tasks differ, and the former ones are more complex as depicted in Figure 8. In turn, impairments in learning CFs may also lead to impairments in social interactions. On the other hand, extreme interest in non-social tasks leads to limited practicing in social ones supporting the idea that E-S theory is part of the Autism Palette. E-S theory is closely related to social motivation theory that follows below.

### Social Motivation Theory

Chevallier et al. (142) suggested that impaired social cognition originates from impaired social motivation. In their model, social motivation integrates three forms of social behavior: social orienting, seeking-liking, and social maintaining. Due to the relevance and the evolutionary advantages of social interactions, and to the higher complexity of such interactions, we believe that social motivation is an evolutionarily emerging characteristic supported by genetic features promoting learning in the social domain. The following section on genetics provides further support about the connection between impaired social motivation and autism including the biological mechanisms that promote social motivation.

### Summary of This Section

In this section, we proposed ways using our framework that show the merits of previous theories and help to overcome some of their limitations and weaknesses mentioned in the literature. We gave a possible explanation for the IQ distribution in autism. We also proposed a solution to overcome the contradiction between the top-down nature of WCC (133) and the low-level sensory processing features of autism described by Robertson and Baron-Cohen (134). The lack of explanation of the presence of comorbidities in the Bayesian Prior Theory (135) can be resolved in our framework. The possibility of an inner orientation to empathizing (140) and the Social Motivation Theory (142) support the social specific reinforcement component of our framework. In section Vulnerabilities we considered “Mirror Neurons” and that CF1s offer a natural explanation to their apparent behavior. We elaborate on the explanatory power of our proposal regarding the controversies of the Mirror Neuron Theory (43, 143, 144) arising from neuronal responses to self-motion and to the motions of others in the Supplementary Materials.

## Genetics

Every living system has access to vast amounts of sensory information. The selection of some useful features—i.e., higher order spatio-temporal correlations—can be vital for reacting to environmental changes. We call these spatial, or temporal, or spatio-temporal features CFs. In a given situation, some of them are relevant for the individual, whereas others are to be neglected. Extraction of the CFs are supported by neuronal sub-architectures and specific mechanisms, such as the procedures of the entorhinal-hippocampal complex, being responsible for episodic memory in mammals. Relevant CFs are supported by success and failures, that is, by means of internal and external reinforcing signals.

Dupre and Yuste (145) showed that even hydras, a genus of cnidarians, are capable of reducing the inputs from the outside world to four components. The manipulation and combination of these components enable them to carry out some basic behaviors. Neuronal circuitries and their building blocks, i.e., the molecules and genes have great similarities from hydras to humans. In the neural network of the hydra, the four components work as four distinct clusters of co-activated neurons. These clusters are anatomically non-overlapping functional circuits and the neurons interact with each other by sodium, potassium, and calcium channels and receptors for glutamate, GABA, dopamine, and other conserved peptides. The dissection of behaviors and the underlying circuits is also a relevant issue in humans. In this section, we review some ASD related molecular findings and connect genetic variants with neuronal circuits and behavior.

Based on findings in the literature, we consider whether the effect of genetic impairments on general cognition and on social specific reinforcements can be dissociated in autism. If such separation is possible then it may help in developing patient specific therapies.

### Polygenic Model of ASD

Recent results show that the development of ASD on the molecular level is mostly determined by genetics and partially by epigenetics. Twin studies estimate ASD heritability to be ~80–90% with additional small, but impactful environmental influence (146–148). Genetics also supports our view about the heterogeneous sources of ASD; autism is a complex disease (149). In contrast to Mendelian diseases, where a single causative variant in a single gene can be pinpointed, complex diseases are the outcome of a plethora of variants with variant effect sizes. Based on genetic linkage and genome-wide association studies (GWAS) the number of ASD candidate genes is around 1,000 (150). These genes have various functions and expressional patterns dependent both on tissue type, condition, and developmental phase. Furthermore, genetic variants found in GWAS are mostly non-coding variants (151) with small influences on autism pathogenesis. Genetic variants have variable penetrance, most of them being low or unknown (152). In about 90–95% of the ASD population the causative variants are unknown or predicted to be common, inherited variants.

These findings support the polygenic risk model, where the development of the disease is the result of the combination of genes with small individual effects on ASD pathogenesis. In these cases, there are thousands of causative genetic variants and several hundreds of affected genes (153).

Approximately 5% of the cases are monogenic forms of ASD (82), where a single genetic source causes the disorder. However, such genetic variants are rarely inherited, mostly *de novo* and occur on the chromosomal level, thus affecting several genes at the same time. A large portion of the monogenic cases are among the syndromic forms of ASD, where the number of candidate genes can range from a single gene to hundreds of genes (154). Among these genes pleiotropic genes—i.e., genes that can influence multiple traits or diseases (155)—are frequently involved.

Thus, the heterogeneity of ASD can be explained by polygenicity and the pleiotropic nature of ASD candidate genes (156). Polygenicity and pleiotropy support frequent comorbidities with other psychiatric and neurological diseases.

### Genetics Could Help to Dissociate Domain-general and Social Specific Impairments

Integration of the genetic findings to our framework can explain some of the important features of our model (Figure 7B). First, we discuss the type-dependent effect of genetic variants on comorbidities, especially on ID as they can shed light on some domain-general impairments. In the second part, we consider the domain-specificity of social reward and propose that it could serve as an explanation for the female protective model.

#### Comorbidities With Domain-general Impact Associated With Specific Types of Genetic Variants

Severity of ASD is highly dependent on the manifestation of comorbid conditions such as macrocephaly, epilepsy, schizophrenia, and ID (104, 157–159). These comorbidities are common in ASD and through impairing intelligence they are influencing ASD ethology. However, a minor, but notable portion of autistic individuals does not have any severe comorbidity. Concerning IQ, about one quarter of the ASD cases have severe ID, while half of the cases have normal or above average IQ (159).

We hypothesize that ASD risk increasing genetic variants have small to medium influences, but on many vulnerability pathways, creating a disease-causing combination of impairments (160). To study the relation between genetics and diseases we should separate the types of the causative genetic variants. The ratio of rare, disruptive alleles is higher in severe, than in mild ID, while inherited common variants are more strongly connected to mild, than to severe ID (161). Moreover, ASD-related, rare, disruptive variants are purified by negative selection, but common variants are under positive selection (162). Interestingly, these latter variants are positively associated with intelligence (163). These results suggest that although common ASD variants can be evolutionarily beneficial, cognition decreasing, rare variants are under negative selection.

Strong impairment of component formation can severely impair cognitive abilities in general. These impairments might be partially compensated in less complex tasks via increased interest in those tasks. Certain *de novo* variants can cause severe IDs and give rise to autistic-like symptoms even without impairments in social rewarding. Such cases may exhibit severe comorbidities, e.g., ID and/or epilepsy (164–166). ID can be the consequence of epilepsy, abnormal brain development, severely damaged synaptogenesis, and there are some highly penetrant variants behind these neurological problems, among other ones. Severe damages in the *mTOR* pathway or loss of *SHANK3* demonstrate these cases (see “Molecular Pathways and Neural Circuits as Vulnerabilities” section of the Supplementary Materials).

These thoughts imply that ASD subgroups could be identified according to their underlying impairments. Genetics and the type of genetic variants could help to find single impairments, while comorbidities are informative about the possible sets of associated impairments.

#### Stronger Social Rewarding in Women Justifies the Female Protective Model

As suggested above, the type of causing genetic variants can have a stratificational value on clinical phenotypes. In case of ASD with severe ID, *de novo* variants are more frequent, while in high-functioning ASD cases, inherited common variants can be observed. These inherited variants and family history of psychiatric disorders are positively correlated with IQ. It is striking that this stronger familial influence is observable only in high functioning male patients (167), whereas in female cases, comorbidities, *de novo* variants and rare inherited variants with loss-of-function effects are enriched. Therefore, lower-functioning cases and female cases have a stronger influence from sporadic genetic variants, while high-functioning male cases are influenced more likely by inherited common variants (167).

A recent study tested if shared variants contribute to the disorder by using a standard measure of genetic relation. They compared ASD individuals with unrelated discordant siblings, i.e., unrelated probands and their unaffected siblings. According to the genetic metric, affected individuals were more similar to the affected than to the unaffected member of the unrelated sibling pair (168) as expected. However, common variants and less common, non-coding regulatory variants of dosage sensitive ASD-related genes are inherited more likely from the father (169), while rare, disruptive, coding variants of these genes are mostly transmitted from the mother (170). A possible explanation to this is that variants with moderate effects can be balanced both in man and woman, while variants with larger effects are carried more likely by the mother because of the “female protective model” (171).

Genetic variants of OXT receptors provide further information from multiple points of view. Beyond their effect on prosocial behavior, they show sex-dependent effect on ASD etiology (172). Results suggest that social interaction is more rewarding for women than for men (173–175). Internal motivation for being in social situations helps one to gain skills in the social context. Social rewarding is stronger in females that may be one of the reasons for the “female protective model.” Higher burden of mutations and increased ratio of ID in female ASD cases may be due to the social reward system that compensates or possibly overcomes the effect of mild impairments of cognition related social skills.

Strengthening of this internal reward system could be an effective therapy for individuals with impaired social reward systems. Furthermore, if these therapies are used early, they could work alike to the case of female protection and could be beneficial for individuals with mild developmental delay by increasing their interest and learning efforts in the social domain. Genetic findings suggest that this type of therapy would be the most effective for patients without rare, disruptive genetic variants, and severe comorbidities. Therefore, experimental studies where social rewarding measurements are correlated with genome-wide genetic data seem justified especially when considering that better means of assessment of social motivation are also needed.

### Summary of Section Genetics

In this section, we reviewed the genetic bases of autism and presented cases where genetic variants can be connected to phenotypical changes. We showed that different types of genetic variants can have different effects on autism. This supports our Autism Palette hypothesis. Due to their distinct genetic background, we considered the possibility of dissecting (a) domain-general regularization and (b) social specific reinforcement as two colors of the palette. We also suggested aiming for different therapeutic approaches for patients with different impairments.

## Combining the Arguments

We posed the following question: How come that (a) many subsets of bountiful discrepancies can give rise to a single leading symptom, namely, the impairment in social behaviors and (b) discrepancies may give rise to the leading symptom no matter if some components of those discrepancies are stronger or weaker than usual or even if they are missing?

We have argued that

a) Social interactions as opposed to typical non-social activities involve a larger number of hidden variables of the social partners and are complex. They require highly efficient component discoveries and learning of both separable components, such as the elements of the cloth (i.e., Type 1 Cartesian Factors) and modifiable, but non-separable components, such as color, scent, mood (i.e., Type 2 Cartesian Factors).
b) The searches for adequate behavioral responses in social interactions are to be learned and the solutions to the learning problem should be deduced from experiences and motivational and external reinforcers. Social interactions become harder if motivations are negligible and practicing is infrequent, if specific signs in social interactions are not recognized or misunderstood.
c) Given (a) and (b), there are many potential causes for autism and a few of them may be sufficient to impair social interaction. The set of *potential dysfunctions forms the autism spectrum*, whereas the *combinations of elements of the set form* the *Autism Palette*. The more the number of causes, the less impairments may be sufficient to sum up and to have an impact on social interactions.
d) Genetics reinforces our arguments, since many different genetic causes contribute to autism and they act along different pathways and in diverse ways. Genetics thus shows a large set of potential causes and GWASes indicate that ASD individuals have diverse subsets of genetic variants from an even larger set of putative ASD related variants.
e) Impairments combined in autism include (a) component related impairments, including searching, finding, and consolidating components, encoding the components into the neocortex, manipulating, and controlling the components by means of high precision synchronization either in the virtual cognitive space or in real space, or in both, (b) the reward system for social interactions. This is in line with findings of Warrier et al. (176) on the genetic dissociability of social and non-social (“systemizing”) traits of ASD that translates to our model as follows: (i) social impairments could be the consequence of abnormal reinforcement, or to a somewhat impaired component formation, or both, (ii) ID can be related to severe impairments of component formation, and (iii) systemizing behavior seems to be result of specific motivations. In addition, a stronger internal reward, i.e., motivation for social interaction can compensate social impairments and may support the female protective model.

Although certain aspects of autism may be modeled as illustrated by the predictive, group-structured and sparse autoencoding architecture of Figure 6, the modeling of the diverse features of the potential colors of the Autism Palette remains a major challenge. This weakness is due to the core of our hypothesis that autism is the result of insufficient counteraction to the “course of dimensionality” during cognitive and emotional development and its critical modeling is beyond present capabilities. In our view, social interactions require one to deal with a large number of variables that are (a) well-hidden, (b) may also undergo quick changes, and that (c) their estimation or recognition can be impaired in many ways.

Finally, we note that the diverse origins of the autistic trait suggest that different treatments may be optimal for people with different impairments. Our framework could help in finding the patient-specific mixture of impaired vulnerabilities, i.e., the colors of The Autism Palette, and the related biological mechanisms. For example, increasing the frequency of social interaction together with the employment of external immediate rewards could enhance social motivation and may help the condition of patients with damaged social-specific reinforcement. Similarly, for an efficient cognitive therapy and/or medication, it seems advantageous to determine the individual combinations of impairments in this heterogeneous disorder.

## Author Contributions

ÁF contributed the most to genetics. AL to neuroscience related information and computational principles. LS harmonized this multi-disciplinary writing and provided detailed information about autism related questions. All authors contributed to the article and approved the submitted version.

## Conflict of Interest

LS receives consulting fees and research support from F. Hoffman- La Roche, royalties from Hogrefe Public and has equity interest in Argus Cognitive Inc. AL has equity interest in Argus Cognitive Inc. The remaining author declares that the research was conducted in the absence of any commercial or financial relationships that could be construed as a potential conflict of interest.

## Footnotes

^1^From the point of view of computations, variables not directly sensed are hidden. For example, variables, such as the outputs of light sensors or auditory sensors form the input variables, whereas edges, faces, phonemes, or words are hidden variables.

^2^https://www.audacityteam.org

^3^The mechanism seems very general: triangular neuronal firing emerges in the neocortex during the learning of a cognitive task with a hidden 2-dimensional structure.
