# Supplementary material for: The Autism Palette: Combinations of Impairments Explain the Heterogeneity in ASD
Source: Front Psychiatry. 2020 Dec 2;11:503462. doi: 10.3389/fpsyt.2020.503462 (PMC7738611; doi:10.3389/fpsyt.2020.503462)
Supplement: Supplementary file 1 [file Data_Sheet_1.pdf]

Supplementary Material

This Supplementary Materials contain information in support to our framework that – due to limitations in length – we could not include into the manuscript.

Suggested literature for autoencoders

The concept of autoencoders is critical for our framework. It has an encoding and a decoding arm. The former is called downstream processing, whereas the latter is called upstream processing in the literature on neuroscience.

Available literature on the topic is extensive; we only point out a few representative references: Hinton, et al. (1995) developed an encoding and decoding model that did fit concepts related to information processing in the brain. Olshausen and Field (1996) put forth a sparse coding method for modelling neurons in the primary visual cortex. High performance autoencoder was proposed by Kingma and Welling (2014).

Predictive autoencoder

Internal representations can be predictive: time series of the internal representation (up to time t+1 in Fig. 2B) can compute an estimation for the representation for the next time step (time t+2 in the illustration) without the corresponding input. Such dynamics is sometimes called temporal propagation of the representation. Temporal propagation can be very specific: decoding of the propagated representation may be able to produce a faithful and timely match to the sensory information despite processing delays (Fig. 2B). In such cases, the internal dynamics is said to mimic ongoing sensory processes, which defines the predictive autoencoders. Decoding alone, i.e., when a predictive autoencoder runs in the absence of sensory input is a model for dreaming and it plays a role in memory consolidation in neuronal processes see, e.g., Maingret et al., (2016) and in deep learning, see, also Goodfellow et al., (2014), Hoshen et al., (2016), and Hu et al., (2018).

Details on compression

The smaller the number of representational units having non-zero activations, the better (i.e., the more probable) the internal model of the input space is, provided that the quality of decoding is preserved. This is the so-called Occam's Razor Principle of information theory (Cover and Thomas, 2012) saying that "The simplest explanation is best". Sometimes, concepts of 'word' and 'dictionary' are used to illustrate how the sparse internal representation helps to 'make sense' in space and time by approximating the input (i.e., sentences within the context of this analogy) (see, e.g., Lőrincz et al., 2002). A single element of the representation (i.e., a single decoding unit, or a component of the representation vector) is 'a word' (i.e., a memory item). The full set of the contents of the decoding units is called 'dictionary'. This is the set of all memory items, see, e.g., Bengio et al. (2009) and Jenatton et al. (2011). Contents belonging to indices may represent episodes. In this case, 'making sense' means a spatio-temporal model that can predict.

Notes on sparse coding

In the case of sparse coding, internal representation has indices with non-zero activity values and with zero activities. Indices with non-zero activity values represent the components of the actual input, whereas indices with zero activity values represent the components that are absent from the input. Contents of the active indices (i.e., the words) are combined by (non-)linear additive processes to provide

an estimation of the input, called the decoding of the representation. In the case of the face, a linear matrix can produce a faithful representation (see, e.g., (Makhzani and Frey, 2015)), whereas in the case of natural language processing the representation can produce translated sentences by non-linear transformations (see, e.g., Vaswani et al., 2017).

Recognition by components (Biederman, 1987) and holistic recognition help to understand the concept of 'words' and 'dictionary' and the hierarchical nature of the component structure. For example, faces can be recognized holistically and through their components, see, e.g., the issue, edited by Watson and Robbins (2014) devoted to this subject in Frontiers of Psychology. Combined recognition is advantageous, since holistic recognition is faster, whereas component-based recognition is more robust.

More details on Type 2 CFs

We add the perception of the three-dimensional (3D) nature of the world, the feeling of acceleration, our emotions and pains of different kinds. Although the concept of qualia is a controversial concept (Kind, 2001), it may help to clarify the differences between CF1s and CF2s that we list below:

- We have access to CFs via our sensory-motor system
- CF1s can be added/coupled, may interact and are decomposable components.
- CF2s do not exist by themselves, they are restrictive (product-like), they can be modified. CF2s are non-decomposable components.
- There are CFs that may correspond both to CF1s and CF2s; the two types of our Cartesian Factors are not exclusive. For example, the stripes on a horse-like creature can be interpreted as both CF1 (at the pigment level) and CF2s (as colors).
- Within the subspace of a CF2, sometimes it is useful to develop a metric. For example, the wavelength of a single color can be determined. Colors may combine multiple single colors, e.g., white color is made of a continuous spectrum. In turn, for any color, further decomposition may be possible by gratings or prism. CF1s can have CF2 components (like a car can have color and shape), and CF2s may be made of CF1s (like the white color of a car is made of many CF1 color components).

Frankish (2016) relates qualia to illusionism. We think that illusions support the concept of both types of CFs. For example, additive component-like form may play a role in the well-known Kanizsa illusion: short line intervals defined by PacMan-like figures are connected by the illusion and we 'see' an object surrounded by these extended intervals in a somewhat different color, although the color is the same as in its immediate neighborhood (Fig. 3B): parts are combined by our brain.

Another illusion is qualia-like and is shown in Fig. 3C. Ends of line segments define a circle and the full disk defined by this circle gains the illusory color of the edges although the background is white. Possibly, color and shape are encoded by separate channels, namely by color and shape boundaries, and decoding overruns sensory information giving rise to color spreading and a modified percept (Pinna and Grossberg, 2005).

AI approaches for Type 2 CFs

Considerable efforts have been directed towards Type 2 CFs in the field of Artificial Intelligence (AI), recently. In the context of deep learning, the effort is called disentanglement. For example,

representations separate writing styles and letters, faces and facial expressions, car types and orientation of the cars see, e.g., Achille and Soatto (2018), Gabbay and Hoshen (2020), and the references therein.

Reinforcement learning

The problem of temporal credit assignment (Sutton, 1984) is the subject of reinforcement learning. This learning method is efficient if the number of states is limited and if all information about the past and present are available, that is, if the state is Markov. Both can be questioned in social tasks, since variables of the states are hidden and their number that enters the exponent of the state space is large. In addition, the structure of rewards is crucial, but seems to be aberrant in ASD both in social and nonsocial aspects (Clements, 2018).

Note that reinforcement learning has different meanings to different communities. Our usage corresponds to goal-oriented behavior and its optimization. The interested reader is referred to the newest edition of the classical book of Sutton and Barto (2018) on this matter. Reinforcement that guides the optimization of behavior is typically delayed and the algorithmic arsenal needs to solve the credit assignment problem. In addition, (a) reinforcers can be either external or internal, including the social domain and (b) their nature can be direct e.g., food or money and indirect or social, such as the smile or the anger of another person.

As an interesting example, Lawson et al. (2017) are concerned with reward learning and the volatility of sensory environment. They find that adults with autism tend to overestimate the volatility of the sensory environment, but – in their design – the learning of rewards concerns the sensory environment and not the learning of the optimization of cumulated and discounted long-term rewards, the subject of reinforcement learning. In turn, care is needed, since their finding is more closely related to feature (CF1 and CF2) extraction, i.e., to the learning of what are the key characteristics of the reward than to the reinforcement learning machinery itself. The same holds for the paper of Crawley et al., (2019)."

Dimension Reduction for Proper Social Behavior: Factored Reinforcement Learning

We argued that solving social behavior tasks is much harder than solving problems in IQ tests, since many variables in social learning and social responses are well hidden. An additional issue is the nature of rewards and that rewards are delayed. Some rewards/penalties, like pain, occur on the millisecond scale, but participating in social interactions could be beneficial only in a longer run. In turn, reinforcement learning, this trial-and-error method should be invoked because of the delays of the rewards and the collection of any "learning sample" needs considerable time because of the durations of the social interactions. Furthermore, reinforcement taxes the learning process, since the complexity of solving such problems is proportional to the number of variables *multiplied by three* (Kearns and Singh, 2002) and this number enters to the exponent.

For factored reinforcement learning (Kearns and Koller, 1999) only the necessary factors are given for each step of the decision making process. That exhibits favorable scaling properties even if there are many steps and the number of all the variables is large (Szita and Lőrincz, 2009). However, the solving of the joined problem, i.e., factor learning *and* behavior optimization, which is needed for the optimization of social interactions is the combination two hard problems and may be impaired in many ways, such as if

social rewards (e.g., smiling) are rewarding, if the interpretation of social rewards is correct (i.e., if facial expressions are properly decoded), if the number of variables is small (i.e., if components are properly formed and the relevant components can be selected), if cognitive manipulations (e.g., risk estimation and planning) are fast and efficient, among others.

Missing information is another serious obstacle according to the theory of reinforcement learning (Krishnamurthy, 2016), since in such cases the actual state becomes uncertain. CF1s can approximate missing information pieces via pattern completions and thus may simplify the learning of behavior optimization. In turn, the learning and selection of CF1s is critical.

A predictive autoencoder architecture

From the point of view of behavior optimization, a serious bottleneck is that at any time instant only a small part of the world is observed, and partial observation can corrupt decision making (Krishnamurthy, 2016). Representations in predictive autoencoders minimize this problem and bring estimations about the uncertainties of future outcomes. Theoretical efforts and computational studies demonstrate the efficiency of this approach (Milacski et al., 2019a, Milacski et al., 2019b). Figure 6 illustrates the complexity of predictive autoencoders that can be viewed as a model of the minicolumnar organization. The figure may serve the interested reader in understanding certain specific aspects of autism modeling. The minicolumnar structure seems to be guided by double-bouquet cells that inhibit cells in other minicolumns (see, e.g., DeFelipe (2011) and the references therein). Furthermore, these cells seem to shape the neocortical structure in primates, but not in other mammalian species (Yáñez et al., 2005)

The large number of adjustable parameters (i.e., weights) and network hyper-parameters, such as the parameters that try to keep all units active (lifetime sparsity) and the parameters that try to save the number of active neurons (spatial sparsity) make modeling effort dubious. Computational studies of one of the authors and his coworkers (Milacski et al., 2019) show that speed and interaction strength between the structures (i.e., the model minicolumns) of the recurrent deep network influence performance in many ways. We hypothesize that these variables can explain the contradictory findings about the distances between minicolumns in ASD (Casanova et al., 2006, McKavanagh et al., 2015).

Mirror Neuron Theory

Since the discovery of the so called mirror neurons (Gallese et al., 1996; Rizzolatti et al., 1996), that react similarly for goal-oriented self-motions and for similar motions of others and thus allow estimations of the intentions of others, it has been considered that the mirror neuron system may be impaired or possibly dysfunctional in autism, see, e.g., (Williams et al., 2001; Rizzolatti and Fabbri-Destro 2010; Hillus et al. 2019, and the cited references therein). It was thought that in typical cases, mirror neurons can provide supervisory information for training and for copying behavioral templates.

Experiments support this assumption to some extent: μ waves are typically blocked or reduced during voluntary muscle movement, e.g., when opening or closing the hands, regardless of whether the subject makes the movement or observes someone else making it, but they were not

blocked even in high-functioning autistic children when they monitored someone else's muscle movement (Oberman et al., 2005).

These findings on μ waves used to be considered compelling (but, in our view, they are only implicit) evidences for problems with the mirror neuron system (Ramachandran and Oberman, 2006). Indeed, contradicting evidence has been found. For example, (Bird et al., 2007; Sowden et al., 2016) showed that automatic imitation is intact in ASD. Furthermore, upon separating automatic imitation from spatial compatibility effects (i.e., separating responses on the same side and on the opposite side), there was no relationship between spatial compatibility and autism symptom severity, meaning that individuals with ASD exhibited increased (and not decreased) imitations. The phenomenon is called hyper imitation (Bird et al., 2007; Spengler et al., 2010; Sowden et al., 2016; Deschrijver et al., 2017). These findings are supported by evidence that individuals with ASD frequently engage in strong imitative behavior, such as echolalia and echopraxia.

We note that component learning should support the development of hand representations making it independent from the owner of the hand to decrease the curse of dimensionality. However, imitation does not require this separation and lacking this separation imitation may become similar to repetition and – intriguingly – that could account for the μ wave-related findings.

We suggest that the formation of Cartesian Factors, e.g., the CF of the hand works as an apparent mirror neuron system. However, it serves dimension reduction instead and other CFs can represent the owner of the hand. In turn, opposed to the assumed supervisory role of mirror neurons, the predictive autoencoder model offers an alternative explanation, the unsupervised learning of CFs that may be misinterpreted as a mirror neuron system. In addition, repetitions are highly useful for unsupervised learning since more learning samples help to eliminate accidental noisy parts of the signals providing further support to our model.

Molecular Pathways and Neural Circuits as Vulnerabilities

The high number of putative genetic variants and the pleiotropic nature of affected genes shifted the interest of geneticists to molecular pathways, where the effect of multiple genes can be integrated, thus the number of causative molecular mechanisms can be reduced (Pinto et al., 2014). In ASD research, pathways of interest include those that 1) regulate cell growth, 2) connect cells to each other, and 3) support communication between them, form circuits and regulate the network of these circuits.

From the point of view of neuropsychiatry, dysfunction in an ASD related pathway may be considered a vulnerability, which in combination with other vulnerabilities can manifest in the development of ASD (Beauchaine and Constantino, 2017). As pleiotropic genes are active in multiple pathways there are also pathways that can influence multiple diseases, as in the case of neuropsychiatric diseases, where shared pathways are frequent (Gandal et al., 2018) and they can be viewed as shared vulnerabilities of comorbid diseases. Further, the severity of ASD is presumed to be dependent on the type of genetic variants and the number of impaired

vulnerabilities (van de Lagemaat and Grant, 2010). For example, genetic variants with cell type or developmental phase specific effects (e.g., non-coding single-nucleotide variants) may have a weaker effect than variants with more general influence (e.g., chromosomal anomalies, copy number variants, likely gene-disrupting mutations).

mTOR Pathway

One of the most studied pathways is the *mTOR* (mammalian target of rapamycin) pathway, which is strongly affected in multiple traits/diseases: e.g. cancer (Efeyan and Sabatini, 2010), epilepsy (Lipton and Sahin, 2014), intellectual disability (ID) (Troca-Marín et al. 2012), ASD (Kelleher and Bear, 2008), and related phenotypes: e.g. macrocephaly (Lee et al., 2012). Mutations in factors of the mTOR pathway dysregulate neuronal translation. Both of the above-mentioned neurological diseases show high overlap with ASD: ~ 30-40% of patients with ASD also have epilepsy (Amiet et al., 2008; Yasuhara, 2010) and they frequently have brain overgrowth (Lainhart et al., 1997). Comorbidity with epilepsy also increases the risk of ID and the prevalence of epilepsy and ID, both being higher in autistic females than males (Amiet et al., 2008). The over-activation of *mTOR* pathway can cause epilepsy through increasing spine density and excitation (Li et al., 2010), but on the other hand it can induce macrocephaly by increasing neuronal size (Crino, 2011). 30-40% of autistic children experience brain overgrowth in their early childhood (Courchesne et al., 2001; Lainhart et al., 1997) and in cases of high-risk children this ratio is even higher (Hazlett et al., 2017). Some ASD candidate genes are implicated in brain size regulation in a dose-dependent manner (Horev et al., 2011). Knowing that *mTOR* pathway regulates several important cell functions such as cell proliferation, synaptogenesis (Crino, 2011), and variants in the regulators of *PI3K-AKT-mTOR* pathway were detected in half of the cases of co-occurring macrocephaly and ID (Yeung et al., 2017), impairment in *mTOR* pathway is the most probable mechanism behind ASD related macrocephaly. Furthermore, loss of *FMR1* (key gene in Fragile X syndrome) also enhances *mTOR* activity (Sharma et al., 2010). Three genes among the early discovered ASD genes (*PTEN* and *TSC1/TSC2*) are repressors of *mTOR* (Inoki, et al. 2005). In *TSC1*/*TSC2* knock-out (KO) inhibition decreases, while in *PTEN* KO both inhibition, excitation, and spine density increase (Gao and Penzes, 2015). Both cases can lead to elevated E/I ratio and epilepsy (Bateup et al., 2013). Altogether, loss of function of either *PTEN* or *TSC1*/*TSC2* enhances *mTOR* activity, but in different ways and with different co-functions. Enhanced *mTOR* activity is a severe impairment with a general effect on brain development and a cause of several overlapping co-morbid outcomes.

Synaptic Impairments Can Disrupt ASD Related Circuits

Besides direct and indirect regulators of neural growth, many ASD candidate genes are factors of molecular pathways with synapse specific functions (Bourgeron, 2009) such as receptors, ion channels, adhesion molecules, and other membrane and scaffold proteins. Excitatory and inhibitory synapses are characterized by different sets of molecules. Pre-synaptic vesicular glutamate transporters (VGLUTs) and receptors (NMDARs and AMPARs) are specific for excitatory synapses. Inhibitory synapses have GABA transporters (VGATs) and receptors (GABAARs and GABABRs) (Eccles, 2013). Their dysfunctions lead to excitatory and inhibitory imbalance, an emerging hypothesis of ASD pathomechanism (Gao and Penzes, 2015). In case of synaptic genes, a neuronal specific form of molecular vulnerabilities, the circuitry becomes

involved. While biochemical pathways (e.g. the previously mentioned *mTOR* pathway) are mainly defined intracellularly, circuits are describing intercellular connections, which are fulfilled through synaptic communication.

Synaptic genes are good examples to show the specific impact of different genes with similar molecular function. *SHANK*s, a family of scaffold proteins, fulfill important adapter functions by anchoring and clustering receptors, ion channels and other membrane proteins on the postsynaptic membrane (Sheng and Kim, 2000). They are among the most studied ASD genes and influencing ASD severity by distinct cognitive impairments. Haploin sufficiency for SHANK3 is considered as the most important genetic cause of the 22q13.3 deletion syndrome (Bonaglia et al., 2001; Wilson et al., 2003), a syndromic form of ASD, also known as Phelan-McDermid syndrome. The major symptom of this SHANK3 related disorder is severe ID (Phelan and McDermid, 2011) (Fig. 7B, left side). However, the other two members of the SHANK family (SHANK1 and SHANK2) are not associated with severe ID. SHANK2 mutations are enriched in ASD individuals with mild ID, while SHANK1 is not associated with ID (Leblond et al., 2014).

Neurexins and neuroligins are important adhesion molecules and strong candidate genes of ASD and other cognitive diseases (Südhof, 2008). They stabilize synaptic connections and shape them by tuning their properties, like their excitatory/inhibitory activities (Chih, et al. 2005). Their function can be very specialized due to their huge number of splicing variants (Ichtchenko et al., 1996). Formation of neuronal circuits is heavily dependent on the precise regulation of alternative splicing of these adhesion molecules (Nguyen et al., 2016).

Here we have reviewed that the diverse genetic backgrounds behind ASD can induce heterogeneity through affecting several neuronal pathways: neuronal growth, synaptogenesis, synaptic plasticity and many others. ASD symptoms and comorbidities could depend on the number and the type of impairments in these pathways. Some autism-related genetic variants as loss-of-function mutations in PTEN and TSC1/2 or SHANK3 deletion can severely damage intelligence as we would expect from domain-general impairments, while other ASD related variants as SHANK1 mutations do not influence intelligence at all. We suggest that ID related variants damage general cognition, while IQ unrelated variants affect reinforcement of social behavior.

References

Achille, A., and Soatto, S. (2018). Emergence of invariance and disentanglement in deep representations. *Journal of Machine Learning Research,* 19(1), 1947-1980.

Amiet, C., Gourfinkel-An, I., Bouzamondo, A., Tordjman, S., Baulac, M., Lechat, P., et al. (2008). Epilepsy in autism is associated with intellectual disability and gender: Evidence from a meta-analysis. *Biological Psychiatry*, *64*(7), 577–582.

Bateup, H. S., Johnson, C. A., Denefrio, C. L., Saulnier, J. L., Kornacker, K., and Sabatini, B. L. (2013). Excitatory/inhibitory synaptic imbalance leads to hippocampal hyperexcitability in mouse models of tuberous sclerosis. *Neuron*, *78*(3), 510–522.

Beauchaine, T. P., and Constantino, J. N. (2017). Redefining the endophenotype concept to accommodate transdiagnostic vulnerabilities and etiological complexity. *Biomarkers in Medicine*, *11*(9), 769–780.

Bengio, S., Pereira, F., Singer, Y., and Strelow, D. (2009). Group sparse coding. Advances in Neural Information

Processing Systems (pp. 82-89).
Biederman, I. (1987). Recognition-by-components: A theory of human image understanding. *Psychological Review*, 94(2), 115.
Bird, G., Leighton, J., Press, C., and Heyes, C. (2007). Intact automatic imitation of human and robot actions in autism spectrum disorders. *Proceedings of the Royal Society B: Biological Sciences*, 274(1628), 3027-3031.
Bonaglia, M. C., Giorda, R., Borgatti, R., Felisari, G., Gagliardi, C., Selicorni, A., and Zuffardi, O. (2001). Disruption of the ProSAP2 Gene in at(12;22)(q24.1;q13.3) Is Associated with the 22q13.3 Deletion Syndrome. *The American Journal of Human Genetics* (Vol. 69).
Bourgeron, T. (2009). A synaptic trek to autism. *Current Opinion in Neurobiology*, *19*(2), 231–234.
Casanova, M. F., van Kooten, I. A., Switala, A. E., van Engeland, H., Heinsen, H., Steinbusch, H. W., ... & Schmitz, C. (2006). Minicolumnar abnormalities in autism. *Acta Neuropathologica*, 112(3), 287.
Chih, B., Engelman, H., and Scheiffele, P. (2005). Control of excitatory and inhibitory synapse formation by neuroligins. *Science*, *307*(5713), 1324–1328.
Clements, C. C., Zoltowski, A. R., Yankowitz, L. D., Yerys, B. E., Schultz, R. T., & Herrington, J. D. (2018). Evaluation of the social motivation hypothesis of autism: A systematic review and meta-analysis. *JAMA Psychiatry*, 75(8), 797-808.
Courchesne, E., Karns, C. M., Davis, H. R., Ziccardi, R., Carper, R. A., Tigue, Z. D., et al. (2001). Unusual brain growth patterns in early life in patients with autistic disorder: An MRI study. *Neurology*, *57*(2), 245–254.
Cover, T. M., and Thomas, J. A. (2012). Elements of information theory. John Wiley and Sons.
Crawley, D., Zhang, L., Jones, E. J., Ahmad, J., Caceres, A. S. J., Oakley, B., ... & Loth, E. (2020). Modeling flexible behavior in childhood to adulthood shows age-dependent learning mechanisms and less optimal learning in autism in each age group, *PloS Biology*, 18(10), p. e3000908.Crino, P. B. (2011). MTOR: A pathogenic signaling pathway in developmental brain malformations. *Trends in Molecular Medicine*, *17*(12), 734–742.
DeFelipe, J. (2011). The evolution of the brain, the human nature of cortical circuits, and intellectual creativity. *Frontiers in Neuroanatomy*, 5, 29.
Deschrijver, E., Wiersema, J. R., and Brass, M. (2017). The influence of action observation on action execution: Dissociating the contribution of action on perception, perception on action, and resolving conflict. *Cognitive, Affective, and Behavioral Neuroscience*, 17(2), 381-393.
Eccles, J. C. (2013). *The physiology of synapses*. Academic Press.
Efeyan, A., and Sabatini, D. M. (2010). MTOR and cancer: Many loops in one pathway. *Current Opinion in Cell Biology*, *22*(2), 169–176.
Frankish, K. (2016). Illusionism as a theory of consciousness. Journal of Consciousness Studies, 23(11-12), 11-39.
Gabbay, A., & Hoshen, Y. (2019). Demystifying inter-class disentanglement. arXiv:1906.11796. International Conference on Learning Representations, 2020
Gallese, V., Fadiga, L., Fogassi, L., and Rizzolatti, G. (1996). Action recognition in the premotor cortex. *Brain*, *119 (Pt 2*(September), 593–609.
Gandal, M. J., Haney, J. R., Parikshak, N. N., Leppa, V., Ramaswami, G., Hartl, C., et al. (2018). Shared molecular neuropathology across major psychiatric disorders parallels polygenic overlap. *Science*, *359*(6376), 693–697.
Gao, R., and Penzes, P. (2015). Common Mechanisms of Excitatory and inhibitory imbalance in schizophrenia and autism spectrum disorders. *Current Molecular Medicine*, *15*(2), 146–167.
Goodfellow, I., Pouget-Abadie, J., Mirza, M., Xu, B., Warde-Farley, D., Ozair, S., ... and Bengio, Y. (2014). Generative adversarial nets. Advances in Neural Information Processing Systems (pp. 2672-2680).
Hazlett, H. C., Gu, H., Munsell, B. C., Kim, S. H., Styner, M., Wolff, J. J., et al. (2017). Early brain development in infants at high risk for autism spectrum disorder. *Nature*, *542*(7641), 348–351.
Hillus, J., Moseley, R., Roepke, S., and Mohr, B. (2019). Action semantic deficits are associated with impaired motor skills in autistic adults without intellectual impairment. *Frontiers in Human Neuroscience*, 13:256.
Hinton, G. E., Dayan, P., Frey, B. J., and Neal, R. M. (1995). The" wake-sleep" algorithm for unsupervised neural networks. *Science*, 268(5214), 1158-1161.
Horev, G., Ellegood, J., Lerch, J. P., Son, Y.-E. E., Muthuswamy, L., Vogel, H., et al. (2011). Dosage-dependent phenotypes in models of 16p11.2 lesions found in autism. *Proceedings of the National Academy of Sciences*, *108*(41), 17076–17081.
Hoshen, Y., Li, K., and Malik, J. (2019). Non-adversarial image synthesis with generative latent nearest neighbors. IEEE Conference on Computer Vision and Pattern Recognition (pp. 5811-5819).
Hu, Z., Yang, Z., Salakhutdinov, R., and Xing, E. P. (2018). On unifying deep generative models. International Conference on Learning Representations arXiv:1706.00550.
Inoki, K., Corradetti, M. N., and Guan, K. L. (2005). Dysregulation of the TSC-mTOR pathway in human disease.

Nature Genetics, *37*(1), 19–24.
Jenatton, R., Mairal, J., Obozinski, G., and Bach, F. (2011). Proximal methods for hierarchical sparse coding. *Journal of Machine Learning Research*, 12, 2297-2334.
Kearns, M., and Singh, S. (2002). Near-optimal reinforcement learning in polynomial time. *Machine Learning*, 49(2–3), 209–232.
Kearns, M., and Koller, D. (1999). Efficient reinforcement learning in factored MDPs. IJCAI International Joint Conference on Artificial Intelligence, 2, 740–747.
Kelleher, R. J., and Bear, M. F. (2008). The autistic neuron: Troubled translation? *Cell*, 135(3), 401–406.
Kind, A. (2001). Qualia realism. Philosophical Studies: An International *Journal for Philosophy in the Analytic Tradition*, 104(2), 143-162.
Kingma, D. P., and Welling, M. (2014). Auto-encoding variational Bayes. International Conference on Learning Representations, arXiv:1312.6114.
Krishnamurthy, V. (2016). Partially observed Markov decision processes: From filtering to controlled sensing. Cambridge University Press.
Lainhart, J. E., Piven, J., Wzorek, M., Landa, R., Santangelo, S. L., Coon, H., and Folstein, S. E. (1997). Macrocephaly in children and adults with autism. *Journal of the American Academy of Child and Adolescent Psychiatry*, *36*(2), 282–290.
Lawson, R. P., Mathys, C., & Rees, G. (2017). Adults with autism overestimate the volatility of the sensory environment. *Nature Neuroscience*, 20(9), 1293.
Leblond, C. S., Nava, C., Polge, A., Gauthier, J., Huguet, G., Lumbroso, S., et al. (2014). Meta-analysis of SHANK Mutations in Autism Spectrum Disorders: A Gradient of Severity in Cognitive Impairments. *PLoS Genetics*, *10*(9).
Lee, J. H., Huynh, M., Silhavy, J. L., Kim, S., Dixon-Salazar, T., Heiberg, A., et al. (2012). De novo somatic mutations in components of the PI3K-AKT3-mTOR pathway cause hemimegalencephaly. *Nature Genetics*, *44*(8), 941–945.
Li, N., Lee, B., Liu, R. J., Banasr, M., Dwyer, J. M., Iwata, M., et al. (2010). mTOR-dependent synapse formation underlies the rapid antidepressant effects of NMDA antagonists. *Science*, *329*(5994), 959–964.
Lipton, J. O., and Sahin, M. (2014). The Neurology of mTOR. *Neuron*, *84*(2), 275–291.
Lőrincz, A., Szatmáry, B., & Szirtes, G. (2002). The mystery of structure and function of sensory processing areas of the neocortex: A resolution. *Journal of Computational Neuroscience*, 13(3), 187-205.
Maingret, N., Girardeau, G., Todorova, R., Goutierre, M., & Zugaro, M. (2016). Hippocampo-cortical coupling mediates memory consolidation during sleep. *Nature Neuroscience*, 19(7), 959-964.
Makhzani, A., and Frey, B. J. (2015). Winner-take-all autoencoders. In Advances in neural information processing systems (pp. 2791-2799).
McKavanagh, R., Buckley, E., & Chance, S. A. (2015). Wider minicolumns in autism: a neural basis for altered processing?. *Brain*, 138(7), 2034-2045.
Milacski, Z. Á., Póczos, B., and Lőrincz, A. (2019a). Group k-sparse temporal convolutional neural networks: Unsupervised pretraining for video classification. In Proceedings of the International Joint Conference on Neural Networks.
Milacski, Z. Á., Póczos, B., & Lőrincz, A. (2019b). Differentiable unrolled alternating direction method of multipliers for OneNet. In: Proceedings of the British Conference on Machine Vision.
Nguyen, T. M., Schreiner, D., Xiao, L., Traunmüller, L., Bornmann, C., and Scheiffele, P. (2016). An alternative splicing switch shapes neurexin repertoires in principal neurons versus interneurons in the mouse hippocampus. *ELife*, *5*(DECEMBER2016), 1–24.
Oberman, L. M., Hubbard, E. M., McCleery, J. P., Altschuler, E. L., Ramachandran, V. S., and Pineda, J. A. (2005). EEG evidence for mirror neuron dysfunction in autism spectrum disorders. *Cognitive Brain Research*, 24(2), 190-198.
Olshausen, B. A., and Field, D. J. (1996). Emergence of simple-cell receptive field properties by learning a sparse code for natural images. *Nature*, 381(6583), 607-609.
Phelan, K., and McDermid, H. E. (2011). The 22q13. 3 deletion syndrome (Phelan-McDermid syndrome). *Molecular Syndromology*, *2*(3–5), 186–201.
Pinna, B., & Grossberg, S. (2005). The watercolor illusion and neon color spreading: A unified analysis of new cases and neural mechanisms. *Journal of the Optical Society of America* A, 22(10), 2207-2221.
Pinto, D., Delaby, E., Merico, D., Barbosa, M., Merikangas, A., Klei, L., et al. (2014). Convergence of genes and cellular pathways dysregulated in autism spectrum disorders. *American Journal of Human Genetics*, *94*(5), 677–694.
Ramachandran, V. S., and Oberman, L. M. (2009). Broken mirrors: A theory of autism. *Scientific American*, *295*(5), 62–69.

Rizzolatti, G., and Fabbri-Destro, M. (2010). Mirror neurons: From discovery to autism. *Experimental Brain Research*, 200(3-4), 223-237.
Rizzolatti, G., Fadiga, L., Gallese, V., and Fogassi, L. (1996). Premotor cortex and the recognition of motor actions. *Cognitive Brain Research*, *3*(2), 131–141.
Sharma, A., Hoeffer, C. A., Takayasu, Y., Miyawaki, T., McBride, S. M., Klann, E., and Zukin, R. S. (2010). Dysregulation of mTOR signaling in fragile X syndrome. *Journal of Neuroscience*, *30*(2), 694–702.
Sheng, M., and Kim, E. (2000). The Shank family of scaffold proteins. *Journal of Cell Science*, *113 (Pt 1)*, 1851–1856.
Sowden, S., Koehne, S., Catmur, C., Dziobek, I., and Bird, G. (2016). Intact automatic imitation and typical spatial compatibility in autism spectrum disorder: Challenging the broken mirror theory. *Autism Research*, 9(2), 292-300.
Spengler, S., Bird, G., and Brass, M. (2010). Hyperimitation of actions is related to reduced understanding of others' minds in autism spectrum conditions. *Biological Psychiatry*, 68(12), 1148-1155.
Sutton, R. S., and Barto, A. G. (2018). Reinforcement learning: An introduction. MIT press.
Südhof, T. C. (2008). Neuroligins and neurexins link synaptic function to cognitive disease. *Nature*, *455*(7215), 903–911.
Szita, I., and Lőrincz, A. (2009). Optimistic initialization and greediness lead to polynomial time learning in factored MDPs. Proceedings of the 26th Annual International Conference on Machine Learning, 1001–1008. ACM.
Troca-Marín, J. A., Alves-Sampaio, A., and Montesinos, M. L. (2012). Deregulated mTOR-mediated translation in intellectual disability. *Progress in Neurobiology*, *96*(2), 268–282.
van de Lagemaat, L. N., and Grant, S. G. N. (2010). Genome variation and complexity in the autism spectrum. *Neuron*, *67*(1), 8–10.
Vaswani, A., Shazeer, N., Parmar, N., Uszkoreit, J., Jones, L., Gomez, A. N., Kaiser, Ł. and Polosukhin, I. (2017). Attention is all you need. In Advances in neural information processing systems (pp. 5998-6008).
Watson, T. L., and Robbins, R. A. (2014). The nature of holistic processing in face and object recognition: Current opinions. *Frontiers in Psychology*, 5, 3.
Williams, J. H., Whiten, A., Suddendorf, T., & Perrett, D. I. (2001). Imitation, mirror neurons and autism. *Neuroscience & Biobehavioral Reviews*, 25(4), 287-295.
Wilson, H. L., Wong, A. C. C., Shaw, S. R., Tse, W. Y., Stapleton, G. A., Phelan, M. C., et al. (2003). Molecular characterisation of the 22q13 deletion syndrome supports the role of haploinsufficiency of SHANK3/PROSAP2 in the major neurological symptoms. *Journal of Medical Genetics*, *40*(8), 575–584.
Yáñez, I. B., Muñoz, A., Contreras, J., Gonzalez, J., Rodriguez-Veiga, E., & DeFelipe, J. (2005). Double bouquet cell in the human cerebral cortex and a comparison with other mammals. *Journal of Comparative Neurology*, 486(4), 344-360.
Yasuhara, A. (2010). Correlation between EEG abnormalities and symptoms of autism spectrum disorder (ASD). *Brain and Development*, *32*(10), 791–798.
Yeung, K. S., Tso, W. W. Y., Ip, J. J. K., Mak, C. C. Y., Leung, G. K. C., Tsang, M. H. Y., Ying, D., Pei, S. L. C., Lee, S. L., Yang, W., and Chung, B. H. Y. (2017). Identification of mutations in the PI3K-AKT-mTOR signalling pathway in patients with macrocephaly and developmental delay and/or autism. *Molecular Autism*, *8*(1), 1–11.
